# Mental health and its association with coping strategies and intolerance of uncertainty during the COVID-19 pandemic among the general population in Saudi Arabia: cross-sectional study

**DOI:** 10.1186/s12888-021-03370-4

**Published:** 2021-07-28

**Authors:** Ahmad N. AlHadi, Mohammed A. Alarabi, Khulood M. AlMansoor

**Affiliations:** 1 Department of Psychiatry, College of Medicine, King Saud University, Riyadh, Saudi Arabia; 2 SABIC Psychological Health Research & Applications Chair (SPHRAC), Department of Psychiatry, College of Medicine, King Saud University Medical City, King Saud University, Riyadh, Saudi Arabia; 3 Self-Development Skills Department, King Saud University, Riyadh, Saudi Arabia

**Keywords:** Depression, Anxiety, Stress, Insomnia, Knowledge

## Abstract

**Background:**

The COVID-19 pandemic has had a major impact on people’s lives globally. The outbreak in Saudi Arabia worsened when the number of cases and deaths rose in March and April of 2020, leading to a national lockdown. This study aimed to assess the factors associated with mental health symptoms in a sample of people residing in Saudi Arabia during the COVID-19 pandemic.

**Methods:**

We conducted an observational cross-sectional study using an online survey distributed via social media, completed by 3032 respondents from all Saudi regions. We collected demographic data, illness history, and scores of validated self-report scales to assess mental health symptoms, intolerance of uncertainty, and coping strategies.

**Results:**

In total, respondents indicated moderate to very severe symptoms during the pandemic as follows: 20.9% for depression, 17.5% for anxiety, and 12.6% for stress. Younger age, female gender, and history of mental illness were associated with higher levels of depression, anxiety, stress, and insomnia. Intolerance of uncertainty and certain coping strategies (such as denial or self-blame) were associated with more severe symptoms.

**Conclusions:**

Mental health is a key concern during the COVID-19 pandemic, especially for the identified vulnerable groups. Agencies concerned with mental health during crises may use the studied associated factors of mental health symptoms to generate targeted policies or interventions.

**Supplementary Information:**

The online version contains supplementary material available at 10.1186/s12888-021-03370-4.

## Background

In March 2020, the World Health Organization (WHO) declared that the 2019 coronavirus disease (COVID-19) can be characterized as a pandemic [1]. COVID-19 is an atypical pneumonia caused by a novel coronavirus that was first detected in Wuhan, China, in December 2019 [2]. The infection spread quickly outside mainland China; by the end of March 2020, its presence was reported in more than 180 countries [1]. Studies conducted during previous viral epidemics have demonstrated their impact on the mental health of people in affected areas. These studies include those conducted during the SARS epidemic [3–7] and the more recent MERS-CoV epidemic [8–11]. Therefore, a pandemic of the magnitude of COVID-19 is likely to have a major impact on mental health, which could lead to higher levels of anxiety, stress, and depression in the entire population [12]. Following the global spread of COVID-19, millions of people started practicing social distancing, and many had to cope with lockdowns that affected their occupational and social needs. Many experts have expressed concerns over the significant economic and health-related consequences of such drastic changes to daily life [13, 14]. Subsequently, numerous studies investigated and documented the mental health burden of the COVID-19 pandemic all around the world [15–17].

People living through the pandemic were struggling with uncertainties regarding COVID-19 itself, including its treatment, spread, control, and how to survive the crisis [18]. For individuals with low risk tolerance thresholds, increasing uncertainty may underlie the worries they experience [19]. In the 1990s, the Laval group developed the Intolerance of Uncertainty (IU) construct [19–21]. Individuals with high IU believe that they are unable to cope with uncertain situations [22]. They are also likely to interpret all ambiguous information as stressful and threatening [23]. Thus, people who cannot tolerate uncertainty may be more susceptible to psychological symptoms during the COVID-19 pandemic. A study during the H1N1 pandemic in 2009 showed that greater intolerance of uncertainty was associated with higher anxiety, as well as increased likelihood of perceiving the pandemic as more threatening [24]. Moreover, when people face stressful situations such as a pandemic, they utilize coping strategies that may or may not be adaptive [25, 26]. In the wake of the SARS epidemic, a study found that certain coping strategies, such as self-blame and denial, were associated with psychiatric comorbidities [4].

The number of COVID-19 cases reported in Saudi Arabia increased rapidly during March and April 2020 [1]. This prompted the Saudi government to take swift action to control the virus’s spread by suspending operations in many government agencies, temporarily closing all schools and universities, suspending public gatherings including inside mosques, and imposing a national curfew [27, 28]. In light of these measures and the situation in Saudi Arabia, our aim in this study was to assess the mental health symptoms of a sample of people in Saudi Arabia, investigate the factors associated with mental health symptoms, and explore the relationship between mental health symptoms and intolerance of uncertainty and coping strategies during the COVID-19 pandemic.

## Methods

### Study design and procedure

This is a quantitative observational cross-sectional study. The current study consisted of 3032 participants from different regions of Saudi Arabia. It was carried from 1 to 9 April 2020 using an online survey distributed through email and social media (mainly Twitter and WhatsApp). We used a snowball sampling strategy and convenience sample selection. Our target population included all male and female Arabic-speakers, aged from 18 to 85 years, and were living in any of the 13 regions of Saudi Arabia during the COVID-19 pandemic.

### Ethics and consent

Informed consent was obtained before starting the survey. Respondents were informed that their participation was voluntary, and that they had the right to withdraw from the study at any time without any obligation to complete the questionnaire. No incentives or rewards were offered. The survey was completed anonymously; no identifiers were collected. The study was approved by the King Saud University Institutional Review Board before data collection (Research Project No. E-20-4789).

### Measures

A self-report online questionnaire was used for data collection. The first part of the questionnaire included participants’ sociodemographic data. The second part comprised questions to measure participants’ knowledge related to COVID-19. The third part contained four validated scales related to mental health. The questionnaire can be found as a supplementary file.

#### Knowledge about COVID-19

After conducting a search of the literature and the Saudi Ministry of Health website, we designed 10 questions to assess respondents’ knowledge about COVID-19, including general knowledge about the disease and specific knowledge about its symptoms and the recommended methods of preventing its spread [29].

#### Mental health symptoms

A common measure of mental health is the Depression, Anxiety, and Stress Scale (DASS-21) [30, 31]. The self-report DASS-21 scale can be filled out online, making it a feasible instrument for use during a period of social distancing and lockdown. We used the Arabic version of the DASS-21 in our study [32]. The scale has 21 items, 7 items for each symptom domain (depression, anxiety, and stress) rated on a 4-point Likert scale ranging from 0 (“did not apply to me at all”) to 3 (“applied to me very much or most of the time”). The total score for each domain or subscale is calculated by adding the scores its items then multiplying sum by 2 to get a score range of 0–42 for each subscale. The resulting total subscale scores are then classified into five severity categories ranging from normal to very severe [30]. We also included an assessment of participants’ sleep; sleep disruption is often associated with higher levels of stress, especially among vulnerable individuals during a stressful situation [33–35]. The Insomnia Severity Index (ISI) is a brief self-report measure of insomnia that can detect insomnia and estimate its severity, and has demonstrated its validity as an online tool [36–38]. We used the Arabic Insomnia Severity Index (ISI) scale, which has adequate validity and reliability [39]. It contains seven items rated on a 5-point Likert scale with a total score ranging between 0 and 28. Higher scores indicate higher insomnia severity.

#### Intolerance of uncertainty scale (IUS)

The original French-language 27-item Intolerance of Uncertainty Scale (IUS) was designed in Quebec, Canada, to assess responses to ambiguous conditions, uncertainty, and future happenings [19]; an English version was later developed and validated [40]. The IUS-12 is a shortened version of the original scale [41] consisting of 12 items rated on a 5-point Likert scale from 1 (“Not at all characteristic of me”) to 5 (“Entirely characteristic of me”). The IUS-12 was translated and adapted to Arabic in a PhD thesis by two of the authors [42]. The Arabic version showed excellent internal consistency, *α* = 0.90, and good test-retest reliability, *r* = 0.89.

#### Arabic brief COPE scale

The Brief COPE scale assesses an individual’s coping choices using 28 Likert-scale items assessing 14 coping strategies [43]. We used the Arabic Brief Cope scale [44] to assess participants’ coping strategies during the COVID-19 pandemic. Response choices range from 1 (“I haven’t been doing this at all”) to 4 (“I’ve been doing this a lot”). We analyzed the scores of the 14 coping strategies in our sample using the FACTOR standalone program to test for dimensionality and the number of extractable factors using the Parallel Analysis (PA) method. The correlation matrix between these 14 coping strategies had adequate parameters and sample adequacy indexes (determinant of matrix = 0.014, Bartlett’s statistic = 13,010.5, df = 78, *p* < 0.001, and Kaiser-Meyer-Olkin index of sampling adequacy KMO = 0.86). The principal components analysis suggested the presence of two factors which could be extracted from these 14 coping strategies, after excluding the “substance use” coping strategy due to a high standard error. The resulting two factors were labeled “problem-based” (comprised of: acceptance, planning, active coping, positive reframing, religion, and self-distraction) and “emotion-based” (comprised of: denial, self-blame, venting, use of instrumental support, use of emotional support, behavioral disengagement, and humor). We computed composite scores for both problem-based and emotion-based coping factors by adding the mean scores for each strategy (2 items for each of the 13 strategies), and those composite scores were used for further analysis.

### Pilot study

We conducted a pilot study on 10 participants (who were excluded from the final study sample) to test the logistics of conducting the study and the questionnaire’s comprehensibility, and to estimate the time needed to complete the survey. It took an average of 10 min to complete the entire survey during the pilot.

### Data analysis plan

We used the Statistical Package for the Social Sciences (SPSS), Version 21.0 (IBM Corp., Armonk, New York, USA) to analyze the data [45]. We used the person-fit standardized test to identify atypical respondents [46]. For descriptive analysis, the means and standard deviations (SD) were used for continuous variables and the frequency and percentage for categorical variables. The compute command was used to compute the scores of the DASS-21 subscales, as well as the ISI, IUS, and coping composite scores. Significant differences between categorical variables in scale scores were estimated using the independent t-test and the one-way analysis of variance (ANOVA) test as appropriate. We used the bivariate Pearson’s correlation to explore the potential correlations between scale scores. Finally, we used multivariate regression analysis to assess whether mental health symptoms measures (DASS-21 subscales and ISI) were associated with the obtained variables. In all data analysis, a 0.05 level of significance was assumed.

## Results

### Survey respondents

The online survey was completed by a total of 3308 people residing in Saudi Arabia. Through person-fit analysis with cut-off score of Zh ±2.0 [46], we discovered that 276 (8.34%) responses were “atypical”, as demonstrated statistically by random answering or consistently choosing the same answer for different scales [47]. These atypical responses were therefore excluded, while the remaining 3032 responses were included in the analysis. All participants completed the Arabic versions of the DASS-21 scale, ISI, Brief COPE, and IUS. Reliability analysis revealed good internal consistency of all four scales (Cronbach’s alpha > 0.81). The results of descriptive analysis of are displayed in Appendix Table 1.

### Sociodemographic variables

Table 1 displays the descriptive analysis of the respondents’ sociodemographic variables and COVID-19 contact history. The majority of respondents (64.4%) were females. Bivariate analysis revealed that females scored significantly higher than males on all measures of mental health symptoms, including depression, anxiety, stress, and insomnia; they also reported higher intolerance of uncertainty (IU) (*p* < 0.001). The mean ± SD age for the respondents was 38.56 ± 11.64 years, ranging from 18 to 82 years. Younger age groups had significantly higher DASS-21 and ISI scores when compared to older age groups (*p* < 0.001). The most frequently reported marital status was being married (64.3%), followed by being single (28.8%). Respondents who had never married had significantly higher DASS-21 and ISI scores when compared to respondents who were married, divorced, or widowed (*p* < 0.001). Younger respondents and those who had never married were also more intolerant of uncertainty (*p* < 0.001). Results of the bivariate analysis are presented in the Appendix Table 2.

**Table 1:** Sociodemographic Variables of Respondents in Saudi Arabia During the COVID-19 Pandemic. *N* = 3032

Variable	n (%)
**Gender**	
Male	1079 (35.6)
Female	1953 (64.4)
**Age groups**	
18–27 years	596 (19.7)
28–37 years	950 (31.3)
38–47 years	767 (25.3)
48–57 years	501 (16.5)
> =58 years	218 (7.2)
**Marital status**	
Single	874 (28.8)
Married	1949 (64.3)
Divorced	171 (5.6)
Widowed	38 (1.3)
**Region of residence in Saudi Arabia**	
Eastern Region	386 (12.7)
Western Regions	678 (22.4)
Northern Regions	204 (6.7)
Southern Regions	207 (6.8)
Central Region	1557 (51.4)
**Occupation**	
Student	296 (9.8)
Government Sector	933 (30.8)
Healthcare	558 (18.4)
Freelance/Private Sector	533 (17.6)
Unemployed	659 (21.7)
Trade	53 (1.7)
**Contact with patients at work:**	
Yes	606 (20)
No	2426 (80)
**Family member with patient contact at work:**	
Yes	1107 (36.5)
No	1925 (63.5)
**Had contact with a suspected or confirmed COVID-19 case:**	
Yes	138 (4.6)
No	2065 (68.1)
Unsure	829 (27.3)

The respondents’ place of residence in Saudi Arabia is shown in Table 1, organized by region as follows: central region (comprising the provinces of Riyadh and Qassim), western region (Makkah, Madinah, and Albaha), northern region (Tabouk, Ha’il, Aljouf, and Northern Borders), southern region (Asir, Jazan, and Najran), and finally the eastern region. Responses came from all 13 provinces of Saudi Arabia. Analysis revealed that respondents from the central region of Saudi Arabia (51.4%), which includes the highly populated city of Riyadh, scored significantly higher on the depression and stress subscales when compared to respondents from the northern and southern regions (*p* < 0.05). In addition, respondents from the western region, which includes densely populated cities like Jeddah and Makkah, scored significantly higher than respondents from the less populated northern region on both the depression and stress subscales (*p* < 0.05). Regarding occupation, the largest proportion of our respondents were government employees (30.8%), and around a fifth reported being currently unemployed (21.7%). Students and trade workers in our sample had significantly higher DASS-21 scores when compared to all other occupations (*p* < 0.05). In addition, unemployed respondents had higher anxiety scores when compared to freelancers (*p* < 0.05). Respondents who reported working in healthcare (18.4%) did not score higher on the depression or anxiety subscale (*p* > 0.05) but did score significantly higher on the stress subscale when compared to freelancers (*p* < 0.05). As for insomnia severity, students had significantly higher ISI scores than other occupations (*p* < 0.001).

#### Contact history

A fifth of the total sample (20%) reported having contact with medical patients in general at their workplace, and a larger percentage (36.5%) reported family members who made contact with patients at work. These respondent subgroups did not have higher measures of mental health symptoms (*p* > 0.05). Survey respondents were asked whether they had had contact with a suspected or confirmed case of COVID-19. Only 138 (4.6%) respondents reported having had such contact. Interestingly, there was a significant difference in scale scores based on whether the respondent reported having had contact with a suspected or confirmed COVID-19, not having such contact, or being unsure. Respondents who were unsure if they had contact with a suspected or confirmed COVID-19 case scored significantly higher on the DASS-21 when compared to those who reported having no contact (*p* < 0.001). Unsure respondents did not, however, differ significantly from respondents who reported positive contact history (*p* > 0.05). Notably, respondents who were unsure if they had had contact with a COVID-19 case had higher IUS scores (*p* < 0.001).

#### History of illness

Table 2 summarizes respondents’ reported history of chronic illnesses and whether they have been diagnosed with a mental illness. The most frequently reported mental disorders were anxiety disorders (11.2%), depressive disorders (10.5%), and sleep disorders (3.1%). The bivariate analysis revealed that respondents who reported having a diagnosed mental illness scored significantly higher on the DASS-21 and ISI (*p* < 0.001), and had higher IUS (*p* < 0.001). Among those with mental illnesses, significantly higher scores on all measures of mental health symptoms were found in respondents who reported being diagnosed with depressive and anxiety disorders, obsessive compulsive disorder, trauma and stress-related disorders, eating disorders, sleeping disorders, and personality disorders (*p* < 0.010).

**Table 2:** History of Medical or Mental Illness of Respondents in Saudi Arabia During the COVID-19 Pandemic. *N* = 3032.\

Illness History	n (%)
**Chronic Medical Illnesses:**	669 (22.06)
**Diagnosed Mental Illnesses:**	584 (19.26)
Depressive Disorders	317 (10.46)
Anxiety Disorders	339 (11.2)
Neurodevelopmental Disorders	4 (0.13)
Psychotic Disorders	10 (0.33)
Bipolar Spectrum Disorders	22 (0.73)
Obsessive Compulsive Disorder	72 (2.37)
Trauma and Stress-Related Disorders	36 (1.19)
Eating Disorders	28 (0.92)
Sleep Disorders	95 (3.13)
Personality Disorders	31 (1.02)
Other Mental Disorders	81 (2.67)

### Symptom severity

Respondents’ levels of depression, anxiety, and stress during the COVID-19 pandemic were estimated using the cut-off scores of the DASS-21 subscales [30]. Table 3 details the number and percentage of respondents with various levels of symptom severity.

**Table 3:** Frequency of Various Levels of Severity of Depression, Anxiety, and Stress as Assessed by DASS-21. *N* = 3032

Subscale Severity Level	Cut-off scores^a^	n (%)
**Depression Subscale**^**a**^**2 (0–42)**		
Normal	(0–9)	2007 (66.2)
Mild	(10–13)	393 (13)
Moderate	(14–20)	381 (12.6)
Severe	(21–27)	118 (3.9)
Very severe	(≥28)	133 (4.4)
**Anxiety Subscale**^**a**^**2 (0–42)**		
Normal	(0–7)	2303 (76)
Mild	(8–9)	197 (6.5)
Moderate	(10–14)	322 (10.6)
Severe	(15–19)	106 (3.5)
Very severe	(≥20)	104 (3.4)
**Stress Subscale**^**a**^**2 (0–42)**		
Normal	(0–14)	2430 (80.1)
Mild	(15–18)	220 (7.3)
Moderate	(19–25)	182 (6)
Severe	(26–33)	135 (4.5)
Very severe	(≥34)	65 (2.1)

^a^*Based Lovibond, S.H. & Lovibond, P.F. (1995). Manual for the Depression Anxiety & Stress Scales* [30]

### Correlation between mental health symptoms, intolerance of uncertainty, and coping strategies

Table 4 displays the means, standard deviations (SD), and Pearson’s (*r*) bivariate correlation of the study’s scale scores. There was a strong and positive correlation between the three subscales of DASS-21 (*p* < 0.01). There was also a strong and positive correlation between each of the DASS-21 subscales and ISI scores (*p* < 0.01). In addition, IUS scores strongly and positively correlated with all four measures of mental health symptoms (*p* < 0.01). Finally, the emotion-based coping composite scores from the Brief COPE scale correlated strongly and positively with all measures of mental health symptoms and IUS (*p* < 0.01), whereas the problem-based coping scores correlated negatively and weakly with depression, and correlated positively but weakly with IUS.

**Table 4:** Bivariate Pearson’s Correlation Coefficient between Depression, Anxiety, and Stress (DASS-21), Insomnia Severity (ISI), Intolerance of Uncertainty (IUS), and Problem and Emotion-based Coping (Brief COPE). *N* = 3032

	Mean (SD)	Pearson’s Correlation Coefficient (*r*)					
		Depression	Anxiety	Stress	Insomnia Severity (ISI)	Intolerance of Uncertainty Score (IUS)	Positive coping
DASS-21 Depression Score*2	8.01 (8.27)						
DASS-21 Anxiety Score*2	4.69 (6.04)	0.71^*^					
DASS-21 Stress Score*2	9.00 (8.68)	0.81^*^	0.78^*^				
Insomnia Severity Index (ISI) Score.	8.03 (6.21)	0.54^*^	0.50^*^	0.59^*^			
Intolerance of Uncertainty Scale (IUS) Score	29.35 (10.50)	0.56^*^	0.51^*^	0.58^*^	0.45^*^		
Problem-Based coping (Brief COPE) composite score	5.46 (1.79)	- 0.10^*^	0.01	0.03	0.03	0.17^*^	
Emotion-Based coping (Brief COPE) composite score	3.19 (1.43)	0.48^*^	0.48^*^	0.51^*^	0.41^*^	0.50^*^	0.38^*^

* Correlation is significant at p < 0.01 (2-tailed)

### Knowledge about COVID-19

A significantly large proportion of respondents (62.1%) either answered “no” or “I do not know” to the question of whether COVID-19 had a known cause, despite the survey being conducted in early April 2020 when sufficient information on the cause of COVID-19 had become available to the public. Nevertheless, there was no association between respondents’ answers to this question and their DASS-21, ISI, or IUS scores (*p* > 0.05). In addition, a significant proportion (41.8%) correctly answered that someone could become infected with COVID-19 without having direct contact with another person, and these respondents had significantly higher DASS-21, ISI, and IUS scores compared to those who did not believe this was possible (*p* < 0.05). The same was found in the significant proportion (91.2%) of respondents who correctly answered that someone with COVID-19 may experience no symptoms, as they scored significantly higher on all four measures of mental health symptoms and had higher IUS scores (*p* < 0.05). Likewise, respondents who knew that there was no treatment for COVID-19 at the time of the survey (58.2%) had significantly higher DASS-21 and IUS scores (*p* < 0.001). The details of the bivariate analysis are presented in Appendix Table 3.

When we analyzed the association between respondents’ knowledge of COVID-19 symptoms and mental health symptoms, we found that the 34.5% of respondents who knew that losing one’s sense of smell (anosmia) may be a symptom of COVID-19 had significantly higher DASS-21 and ISI scores (*p* < 0.05). Those who had better knowledge of the recommended methods of preventing the disease’s spread occasionally scored significantly higher on the DASS-21. This was found for respondents who reported they do not think wearing medical gloves is a recommended prevention method (*p* < 0.05), and for those who knew not touching one’s face is recommended (*p* < 0.05, for measures of anxiety and stress). On the other hand, respondents who correctly reported that using tissues while sneezing and wearing medical masks are recommended preventive measures scored significantly lower on the DASS-21 subscales (*p* < 0.05).

### Regression models for mental health symptoms during the COVID-19 pandemic

We used multivariate linear regression to explore the factors statistically associated with mental health symptoms during the COVID-19 pandemic. The regression models used all significantly associated variables (*p* < 0.05) from the previous bivariate analysis as independent variables. The independent variables were grouped into blocks for each model, and tested in a series of competing models. Only the models with the highest accuracy are included below. The DASS-21 subscales and ISI scores were set as the dependent variables. For the DASS-21 subscales, the scores were log transformed after adding a constant to deal with their skewed distribution.

#### Regression model for depression

The model is detailed in Table 5 and was statistically significant, explaining 68.4% of the variation between respondents in their depression scores. Female gender was associated with increased depression scores, while an increase in age in years was associated with lower depression scores. Contact history with a suspected or confirmed COVID-19 case was a significant factor: having positive contact or being unsure was associated with increased depression scores. Unsurprisingly, history of being diagnosed with a depressive disorder was associated with an increase in depression scores. Believing COVID-19 has no treatment and knowing that anosmia can be a symptom of COVID-19 were also associated with higher depression scores, whereas incorrectly believing that wearing gloves is a recommended prevention method was associated with lower scores. Higher ISI scores and higher IUS scores were both associated with higher depression scores. Coping strategies were also significant factors: higher emotion-based coping scores were associated with higher depression scores, whereas higher problem-based coping scores were associated with lower depression scores. The model suggests that the respondents’ level of depression during the pandemic was most significantly associated with the severity of their insomnia, their underlying intolerance of uncertainty, and whether they employed problem or emotion-based coping strategies.

**Table 5:** Multivariate Linear Regression Analysis of Depression Scores during the COVID19 Pandemic

	n (%)	B (95% CI)	Standardized Beta	t-value	***p***-value
(Constant)		0.320 (0.255 to 0.385)		9.652	< 0.001
Gender = Female	1953 (64.40)	0.062 (0.041 to 0.083)	0.081	5.814	< 0.001
Age (years)		−0.001 (−0.002 to 0.000)	−0.041	−2.896	0.004
Had contact with suspected or confirmed a COVID-19 case = Yes/Unsure	967 (31.89)	0.033 (0.012 to 0.054)	0.043	3.128	0.002
History of depressive disorders = Yes	317 (10.46)	0.094 (0.060 to 0.127)	0.078	5.525	< 0.001
History of trauma and stress-related disorders = Yes	36 (1.19)	0.061 (−0.028 to 0.151)	0.018	1.340	0.180
History of sleep disorders = Yes	95 (3.13)	−0.083 (− 0.140 to − 0.026)	−0.040	−2.863	0.004
Believes anosmia is a COVID-19 symptom = Yes	1045 (34.47)	0.027 (0.007 to 0.048)	0.035	2.621	0.009
Believes wearing medical gloves prevents the spread of COVID-19 = Yes	1562 (51.52)	− 0.023 (− 0.042 to − 0.003)	−0.031	−2.230	0.026
Believes using tissues when sneezing prevents the spread of COVID-19 = Yes	2594 (85.55)	−0.017 (− 0.045 to 0.011)	−0.016	−1.180	0.238
Believes there is a treatment for COVID-19 = No	1765 (58.21)	0.021 (0.001 to 0.040)	0.028	2.062	0.039
Insomnia Severity Index (ISI)		0.017 (0.015 to 0.019)	0.288	17.936	< 0.001
Intolerance of Uncertainty Scores (IUS)		0.010 (0.008 to 0.011)	0.274	16.818	< 0.001
Problem-based coping composite score (Brief COPE)		−0.039 (−0.047 to − 0.030)	−0.140	−9.267	< 0.001
Emotion-based coping composite score (Brief COPE)		0.100 (0.087 to 0.114)	0.247	14.502	< 0.001

*Dependent Variable: Log*_*10*_ *(DASS-21 Depression Subscale Scores + 2), Model statistical significance: F (14, 3016) = 189.14, p < 0.001, R*^*2*^ *= 0. 684, R*^*2*^*Adjusted = 0.465*

#### Regression model for anxiety

Table 6 shows the results of the regression model for anxiety scores. The resulting model was statistically significant, explaining 65.4% of the variation in the anxiety subscale scores between our respondents. As with depression, female gender was associated with higher anxiety, and increasing age in years was associated with a decrease in anxiety. Being married, divorced, or widowed was associated with significantly higher anxiety scores when all other variables were held constant. In addition, having history of contact with a suspected or confirmed COVID-19 case or being unsure was associated with higher anxiety scores. The model also suggested that history of a chronic medical illness and history of an anxiety disorder were both associated with significantly higher anxiety scores. Respondents’ knowledge that diarrhea is a symptom of COVID-19 was associated with higher anxiety; while falsely thinking that wearing gloves is a recommended prevention method was associated with lower anxiety scores. Higher ISI scores were associated with higher anxiety scores, as were higher IUS scores and the composite scores of emotion-based coping. Higher problem-based coping scores, however, were associated with lower anxiety. Therefore, similar to depression, the respondents’ level of anxiety during the COVID-19 pandemic was most importantly associated with the severity of their insomnia, their underlying intolerance of uncertainty, and whether they utilized problem or emotion-based coping strategies.

**Table 6:** Multivariate Linear Regression Analysis of Anxiety Scores during the COVID19 Pandemic

	n (%)	B (95% CI)	Standardized Beta	t-value	***p-***value
(Constant)		0.145 (0.086 to 0.205)		4.790	< 0.001
Gender = Female		0.046 (0.025 to 0.066)	0.063	4.417	< 0.001
Age (years)		−0.002 (− 0.003 to − 0.001)	−0.054	− 2.948	0.003
Married, Divorced, or Widowed = Yes	2158 (71.17)	0.030 (0.005 to 0.055)	0.040	2.353	0.019
Had contact with a suspected or confirmed COVID-19 case = Yes/Unsure	967 (31.89)	0.053 (0.032 to 0.073)	0.072	5.056	< 0.001
History of chronic medical illness = Yes	669 (22.06)	0.039 (0.015 to 0.062)	0.047	3.236	0.001
History of depressive disorders = Yes	317 (10.46)	0.027 (−0.007 to 0.061)	0.024	1.578	0.115
History of anxiety disorders = Yes	339 (11.18)	0.070 (0.037 to 0.103)	0.064	4.201	< 0.001
Believes diarrhea is a COVID-19 symptom = Yes	975 (32.16)	0.024 (0.004 to 0.044)	0.033	2.381	0.017
Believes wearing medical gloves prevents the spread of COVID-19 = Yes	1562 (51.52)	−0.032 (− 0.051 to − 0.014)	−0.047	−3.392	0.001
Insomnia Severity Index (ISI)		0.014 (0.012 to 0.016)	0.252	15.253	< 0.001
Intolerance of Uncertainty Scores (IUS)		0.008 (0.007 to 0.009)	0.249	14.739	< 0.001
Problem-based coping composite score (Brief COPE)		−0.030 (−0.038 to − 0.022)	−0.115	−7.388	< 0.001
Emotion-based coping composite score (Brief COPE)		0.103 (0.089 to 0.116)	0.268	15.177	< 0.001

*Dependent Variable: Log*_*10*_ *(DASS-21 Anxiety Subscale Scores + 2), Model statistical significance: F (13, 3017) = 173.65, p < 0.001, R*^*2*^ *= 0.654, R*^*2*^*Adjusted = 0. 426*

#### Regression model for stress

Table 7 details the results of the regression model for the DASS-21 stress subscale. The model was statistically significant and could explain 73% of all the variation in the stress scores of respondents. As with the other two subscales of DASS-21, female gender was associated with higher stress, while increasing age in years was associated with lower stress. Another sociodemographic factor was being married, divorced, or widowed, which was associated with higher stress scores. Contact history with a suspected or confirmed COVID-19 case was a significant factor as well: having positive contact or being unsure was associated with increased stress scores. History of a depressive disorder was associated with higher stress, as with depression. Knowing that anosmia can be a symptom of COVID-19 was associated with increased stress; mistakenly believing that wearing medical gloves was a recommended prevention method was associated with lower scores. As with depression and anxiety, the most important factors for stress during the COVID-19 pandemic were the severity of insomnia, underlying intolerance of uncertainty, and the use of emotion-based coping strategies—these all were associated with higher stress; in contrast, the use of problem-based coping strategies was associated with lower stress.

**Table 7:** Multivariate Linear Regression Analysis of Stress Scores during the COVID19 Pandemic

	n (%)	B (95% CI)	Standardized Beta	t-value	***p***-value
(Constant)		−5.429 (−6.78 to −4.078)		−7.878	< 0.001
Gender = Female	1953 (64.40)	1.250 (0.785 to 1.716)	0.069	5.272	< 0.001
Age (years)		−0.032 (−.056 to − 0.009)	−0.043	−2.741	0.006
Married, Divorced, or Widowed = Yes	2158 (71.17)	0.692 (0.117 to 1.267)	0.036	2.359	0.018
Had contact with a 19 suspected or confirmed COVID- case = Yes/Unsure	967 (31.89)	1.154 (0.689 to 1.620)	0.062	4.862	< 0.001
History of mental illness = Yes	584 (19.26)	0.603 (− 0.146 to 1.352)	0.027	1.580	0.114
History of depressive disorders = Yes	317 (10.46)	1.778 (0.827 to 2.729)	0.063	3.666	< 0.001
Believes joint pain is a COVID-19 symptom = Yes	1300 (42.88)	0.378 (−0.061 to 0.817)	0.022	1.688	0.092
Believes anosmia is a COVID-19 symptom = Yes	1045 (34.47)	0.562 (0.105 to 1.019)	0.031	2.410	0.016
Believes wearing medical gloves prevents the spread of COVID-19 = Yes	1562 (51.52)	−0.690 (−1.117 to − 0.262)	−0.040	−3.163	0.002
Insomnia Severity Index (ISI)		0.453 (0.412 to 0.494)	0.324	21.725	< 0.001
Intolerance of Uncertainty Scores (IUS)		0.250 (0.225 to 0.275)	0.302	19.777	< 0.001
Problem-based coping composite score (Brief COPE)		−0.856 (−1.037 to − 0.674)	−0.131	−9.252	< 0.001
Emotion-based coping composite score (Brief COPE)		2.277 (1.975 to 2.580)	0.236	14.754	< 0.001

*Dependent Variable: Log*_*10*_ *(DASS-21 Stress Subscale Scores + 2), Model statistical significance: F (13, 3017) = 173.65, p < 0.001, R*^*2*^ *= 0.730, R*^*2*^*Adjusted = 0. 530*

#### Regression model for insomnia severity

The linear regression model for insomnia, detailed in Table 8, was statistically significant and could explain 62.3% of the variance in ISI scores in our respondents. Unlike the DASS-21, higher ISI was not associated with female gender. However, increasing age in years was associated with lower ISI, similar to how it was associated with lower depression, anxiety, and stress. History of a chronic medical illness, a depressive disorder, or a sleep disorder were all associated with greater insomnia severity. The only knowledge questions which were associated with insomnia severity were related to perceived recommended methods of preventing COVID-19. Depression and anxiety, as scored by their respective DASS-21 subscales, were significantly associated with an increase in ISI. Higher IUS scores and emotion-based coping scores were also associated with higher ISI. Problem-based coping, on the other hand, was not significantly associated with ISI scores. The results of the regression model indicate that the most important factors associated with insomnia severity during the COVID-19 pandemic were the level of depression and anxiety, along with the respondent’s intolerance of uncertainty, history of a sleep disorder, younger age, and whether they used emotion-based coping strategies.

**Table 8:** Multivariate Linear Regression Analysis of Insomnia Severity Scores during the COVID19 Pandemic

	n (%)	B (95% CI)	Standardized Beta	t-value	***p-***value
(Constant)		2.053 (0.413 to 3.693)		2.455	0.014
Gender = Female	1953 (64.40)	0.180 (− 0.199 to 0.560)	.014	.930	0.352
Age (years)		−0.069 (− 0.086 to − 0.052)	−0.129	−7.983	< 0.001
Occupation		0.111 (−0.020 to 0.242)	0.025	1.665	0.096
Had contact with a suspected or confirmed COVID-19 case = Yes/Unsure	967 (31.89)	0.235 (−0.106 to 0.576)	0.020	1.349	0.177
History of chronic medical illness = Yes	669 (22.06)	0.580 (0.141 to 1.020)	0.039	2.592	0.010
History of depressive disorders = Yes	317 (10.46)	0.876 (0.266 to 1.487)	0.043	2.816	0.005
History of sleep disorders = Yes	95 (3.13)	4.621 (3.598 to 5.644)	0.130	8.857	< 0.001
Believes a person have COVID-19 without having had contact with someone carrying the virus = yes	1268 (41.82)	0.255 (− 0.100 to 0.611)	0.020	1.408	0.159
Believes shortness of breath is a symptom of COVID-19 = yes	2920 (96.30)	0.759 (−0.198 to 1.716)	0.023	1.554	0.120
Believes not touching one’s face prevents COVID-19 = Yes	2621 (86.44)	0.657 (0.105 to 1.209)	0.036	2.332	0.020
Believes using tissues when sneezing prevents COVID19 = Yes	2594 (85.55)	−0.660 (− 1.197 to − 0.123)	−0.037	−2.409	0.016
DASS-21 Anxiety Subscale Score		0.146 (0.104 to 0.188)	0.142	6.782	< 0.001
DASS-21 Depression Subscale Score		0.183 (0.150 to 0.215)	0.243	10.868	< 0.001
Intolerance of Uncertainty scores (IUS)		0.087 (0.066 to 0.109)	0.148	7.996	< 0.001
Problem-based coping composite score (Brief COPE)		−0.098 (−0.253 to 0.056)	− 0.021	−1.248	0.212
Emotion-based coping composite score (Brief COPE)		0.704 (0.445 to 0.963)	0.102	5.335	< 0.001

*Dependent Variable: Insomnia Severity Index (ISI) Scores, Model statistical significance: F (16, 3014) = 119.70, p < 0.001, R*^*2*^ *= 0.623, R*^*2*^*Adjusted = 0. 385*

## Discussion

In this cross-sectional study, we aimed to study the factors associated with the mental health symptoms of a sample of people in Saudi Arabia during the COVID-19 pandemic. Via social media and convenience-based snowball sampling, we collected a large sample of responses with a good response rate. We used person-fit analysis as detailed above to ensure the quality of our collected data. This removed 8.34% of responses but helped exclude possible careless respondents [47]. The age distribution in our sample resembles that of the Saudi population, where people aged 20 to 50 years constitute the largest age group [48]. In addition, we received responses from all 13 formal regions of the country, with the majority being from the densely populated central, western, and eastern regions [49]. In our sample, female respondents outnumbered male respondents (64.4 and 35.6%, respectively), whereas males constitute a larger percentage in the latest population census (57.7% males vs. 42.3% females) [48]. Therefore, only age and location of residence of our respondents, but not gender distribution, resemble the demographic characteristics of the Saudi population.

Our sample of people in Saudi Arabia had moderate levels of psychological symptoms during the COVID-19 pandemic. The percentage of moderate to very severe symptoms were 20.9% for depression, 17.5% for anxiety, and 12.6% for stress. These results are higher than those of the Saudi Mental Health National Survey (SMHNS), which found the prevalence of Major Depressive Disorder to be 6% and the prevalence of Generalized Anxiety Disorder to be 1.9% [50]. The higher percentage of depression and anxiety symptoms in our sample could be a result of the COVID-19 pandemic and the radical changes to daily life following its spread. Conversely, it could be due to differing assessment tools; we utilized self-report scales in our study, whereas investigators in the SMHNS used structured diagnostic interviews. Further, sampling technique may play a role; we used convenience-based snowball sampling in our study, whereas the sampling was randomized in the SMHNS. Nevertheless, our findings are similar to what has been reported in the COVID-19 literature in other countries. In China, a similar study using the DASS-21 and the same cut-off scores reported moderate to severe symptoms of depression in 16.5%, anxiety in 28.8%, and stress in 8.1% of their sample [51]. Another study in Italy that was conducted during the COVID-19 crisis also used the DASS-21, but defined high and very high severity of symptoms as scores one and two standard deviations above average, respectively. The Italian study revealed that high and very high severity of symptoms of depression were found in 32.8%, anxiety in 18.7%, and stress in 27.2% of their sample [52]. Another European study, conducted in Spain, showed that 18.7% of people had depressive symptoms, 21.6% had anxiety symptoms, and 15.8% had post-traumatic stress symptoms [53]. The situation in the United States may be similar; polls have shown that many Americans suffer from stress and anxiety symptoms, with higher percentages ranging from 45 to 62% [54–56].

Our results show that female gender and younger age are associated with depression, anxiety, and stress during the COVID-19 pandemic. These results match recent findings from China and Italy [51, 52]. The previously cited Spanish study also found that being a female or of younger age was associated with psychological symptoms [53]. Contact history with a suspected or confirmed COVID-19 case, or being unsure of contact, was associated with worse mental health outcomes in our sample, similar as in China and Italy [51, 52]. Moreover, our finding that respondents who had previously been diagnosed with a mental illness had more severe symptoms has also been reported in previous studies showing that mental disorders usually worsen during stressful situations like the COVID-19 pandemic [17]. Similar to how measures of depression and anxiety were associated with more severe insomnia in our sample, a recent Greek study found that insomnia during the pandemic was associated with more depressive symptoms, loneliness, and COVID-19-related worry [57].

Our results suggest that accurate knowledge about COVID-19 does not play a protective role against mental health symptoms during the pandemic, and accurate knowledge may at times be associated with worse symptoms. In China, however, having good knowledge about the route of transmission of COVID-19 correlated with lower levels of anxiety [51]. In Spain, receiving sufficient information about the pandemic was a protective factor against symptoms of depression, anxiety, and PSTD [53]. We suggest developing a valid and reliable measure of knowledge about COVID-19 to assess it more accurately and test its relationship with mental health symptoms.

Regarding the role of intolerance of uncertainty (IU) in mental health during the COVID-19 pandemic, our analysis reveals that higher IU correlates strongly with mental health symptoms. Similarly, two groups of Turkish researchers published studies on the relationship between IU and mental wellbeing during the COVID-19 pandemic [58, 59]. Both studies demonstrated how IU mediated the effect of worry or fear on mental health. The previously cited Greek study also found that IUS scores were associated with insomnia in their sample [57]. This may not be surprising given the evidence that people with high IUS scores respond to uncertain conditions by overestimating the risk and the probability of negative outcomes, therefore increasing their anxiety [21]. During the COVID-19 pandemic, people must face a range of uncertainties, including that of infection, the health status of their loved ones, and the possibility of death [18]. In our sample, people with high IUS scores during the pandemic had higher scores on the DASS-21 and ISI scales, consistent with previous studies suggesting that there is a strong association between IU and depression, anxiety, and insomnia [60–67]. In addition, our study results support the previous finding that intolerance of uncertainty is a trans-diagnostic construct [68–71]. Our analysis also demonstrated that IU correlated strongly with the use of emotion-based coping strategies during the pandemic. This is similar to what was found in a study during the H1N1 pandemic [24]. Our regression analysis suggests that emotion-based coping strategies (such as denial or self-blame) account for some of the depression, anxiety, stress, and insomnia people experienced during the pandemic. A recent Chinese study similarly highlighted the importance of coping strategies during the COVID-19 crisis [72].

This study has multiple limitations. First, our study used a convenience sampling technique which introduces the bias of certain respondents possibly completing the questionnaire more frequently, such as individuals with perceived symptoms or those with an interest in mental health (i.e. selection bias). Second, our results are based on self-report scales for the assessment of mental health symptoms, which limits their reliability. Finally, our results are based on a single cross-sectional assessment.

## Conclusions

Mental health is an important concern during the COVID-19 pandemic in Saudi Arabia. Similar to other countries, people of certain demographics in our sample in Saudi Arabia, such as younger individuals and females, reported more severe symptoms of depression, anxiety, stress, and insomnia during the pandemic. History of mental illness was an important risk factor, highlighting the vulnerability of the mentally ill in times of crisis. Finally, people’s underlying intolerance of uncertainty, and which coping strategies they utilized during the crisis, seem to have been important factors associated with their mental health during the COVID-19 pandemic. Agencies concerned with mental health during crises can make use of the studied associated factors of mental health symptoms to generate policies or interventions that target vulnerable individuals.

## Supplementary Information

**Additional file 1.**

**Additional file 2.**

## Data Availability

The datasets used during the current study are available from the corresponding author on reasonable request.

## Abbreviations

DASS-21: Depression, Anxiety, and Stress Scale (DASS-21)
ISI: Insomnia Severity Index
IUS: Intolerance of Uncertainty Scale
