# Supplementary material for: Mental health and its association with coping strategies and intolerance of uncertainty during the COVID-19 pandemic among the general population in Saudi Arabia: cross-sectional study
Source: BMC Psychiatry. 2021 Jul 28;21:382. doi: 10.1186/s12888-021-03370-4 (PMC8317145; doi:10.1186/s12888-021-03370-4)
Supplement: Supplementary file 1 — Additional file 1. [file 12888_2021_3370_MOESM1_ESM.docx]

| Appendix Table 1: Descriptive statistics of Depression, Anxiety, and Stress (DASS-21) subscales, Insomnia Severity Index (ISI) scores, Intolerance of Uncertainty Scale (IUS) scores, and Brief COPE items score, N=2032. | | | |
| --- | --- | --- | --- |
|  | **Mean** | **SD** | **Maximum possible score** |
| Stress DASS-Stress Score *2. (DASS-21 subscale score multiplied by 2) | 9.00 | 8.68 | 0-42 |
| Anxiety DASS-Anxiety Score *2. (DASS-21 subscale score multiplied by 2) | 4.69 | 6.04 | 0-42 |
| Depression DASS-Depression Score *2. (DASS-21 subscale score multiplied by 2) | 8.01 | 8.27 | 0-42 |
| Insomnia severity Index Score. | 8.03 | 6.21 | 0-28 |
| Intolerance of Uncertainty Total Score (12 items) | 29.35 | 10.50 | 12-60 |
| *Brief COPE Subscales:* |  |  |  |
| COPE-Self-distraction Score. | 4.56 | 1.70 | 2-8 |
| COPE-Active coping Score. | 4.89 | 1.74 | 2-8 |
| COPE-Denial Score. | 2.94 | 1.36 | 2-8 |
| COPE-Substance use Score. | 2.06 | .42 | 2-8 |
| COPE-Use of Emotional Support Score. | 3.60 | 1.63 | 2-8 |
| COPE-Use of Instrumental Support Score. | 3.44 | 1.60 | 2-8 |
| COPE-Behavioral disengagement score. | 2.89 | 1.25 | 2-8 |
| COPE-Venting Score. | 3.48 | 1.37 | 2-8 |
| COPE-Positive reframing Score. | 5.06 | 1.89 | 2-8 |
| COPE-Planning Score. | 5.04 | 1.76 | 2-8 |
| COPE-Humor Score. | 2.96 | 1.32 | 2-8 |
| COPE-Acceptance Score. | 6.22 | 1.72 | 2-8 |
| COPE-Religion Score. | 5.96 | 1.90 | 2-8 |
| COPE-Self-Blame Score. | 3.02 | 1.45 | 2-8 |

|  | Appendix Table 2: The Bivariate Analysis of Depression, Anxiety, and Stress (DASS-21) subscales, Insomnia Severity (ISI), and Intolerance of Uncertainty (IUS) across Sociodemographic variables, contact history, and history of illness | | | | | | | | | | | | | | | | | | | | | | | | | |
| --- | --- | --- | --- | --- | --- | --- | --- | --- | --- | --- | --- | --- | --- | --- | --- | --- | --- | --- | --- | --- | --- | --- | --- | --- | --- | --- |
|  |  | | **Mean (SD)-Stress** | | | **test statistic/df, p-value** | | | | **Mean (SD)-Anxiety** | | **test statistic/df, p-value** | | **Mean (SD)-Depression** | | **test statistic/df, p-value** | | **Mean (SD)-Uncertainty IUS** | | | **test statistic/df, p-value** | | **Mean (SD)-Insomnia** | | | **test statistic/df, p-value** |
|  | **Sex** | |  | | |  | | | |  | |  | |  | |  | |  | | |  | |  | | |  |
|  | Male | | 7.47 (8.01) | | | t(2433.5)=7.5,p<0.001 | | | | 3.94 (5.57) | | t(2443.5)=5.22, p<0.001 | | 6.76 (7.56) | | t(2463.6)=6.50, p<0.001 | | 28.36 (10.19) | | | t(2305.14)=3.90, p<0.001 | | 7.22 (6.00) | | | t(2303.8)=5.43,p<0.001 |
|  | Female | | 9.84 (8.92) | | |  | | | | 5.09 (6.25) | |  | | 8.71 (8.57) | |  | | 29.90 (10.63) | | |  | | 8.48 (6.27) | | |  |
|  | **Age groups** | |  | | |  | | | |  | |  | |  | |  | |  | | |  | |  | | |  |
|  | 18-27 years | | 10.896 (9.29) | | | f(4,1094.04)=57.03, p<0.001 | | | | 5.91 (7.13) | |  | | 10.59 (9.66) | | f(4,1103.7)=55.99, p<0.001 | | 31.85 (10.67) | | | f(4,1067.5)=39.45, p<0.001 | | 10.21 (6.46) | | | f(4,1065.6)=56.4, p<0.001 |
|  | 28-37 years | | 10.99 (9.51) | | |  | | | | 5.77 (6.60) | | f(4,1130.5)=43.32, p<0.001 | | 9.48 (8.94) | |  | | 31.05 (11.14) | | |  | | 8.92 (6.17) | | |  |
|  | 38-47 years | | 8.01 (7.73) | | |  | | | | 4.15 (5.36) | |  | | 6.82 (7.05) | |  | | 28.34 (9.87) | | |  | | 7.35 (6.04) | | |  |
|  | 48-57 years | | 6.12 (6.73) | | |  | | | | 2.95 (4.30) | |  | | 5.49 (6.07) | |  | | 26.69 (9.03) | | |  | | 5.91 (5.30) | | |  |
|  | >=58 years | | 5.19 (6.37) | | |  | | | | 2.53 (3.70) | |  | | 4.60 (5.61) | |  | | 24.77 (9.08) | | |  | | 5.44 (5.28) | | |  |
|  | **Marital state** | |  | | |  | | | |  | |  | |  | |  | |  | | |  | |  | | |  |
|  | Never married | | 10.78 (9.46) | | | t(1662.26)=8.5, p<0.001 | | | | 6.64 (7.8) | | t(1512.3)=7.7, p<0.001 | | 10.35 (9.55) | | t(1329.02)=31.13, p<0.001 | | 31.89 (10.92) | | | t(1515.5)=8.32, p<0.001 | | 9.65 (6.49) | | | t(1503.04)=8.94, p<0.001 |
|  | Married, divorced, or widowed | | 8.72 (8.60) | | |  | | | | 4.15 (5.92) | |  | | 7.10 (7.49) | |  | | 28.32 (10.15) | | |  | | 7.38 (5.97) | | |  |
|  | **Occupation** | |  | | |  | | | |  | |  | |  | |  | |  | | |  | |  | | |  |
|  | Student | | 11.26 (9.70) | | | f (5,460.17)=6.92, p<0.001 | | | | 6.24 (7.50) | | f(5,460.10)=6.41, p<0.001 | | 11.38 (10.01) | | f(5,459.6)=9.73, p<0.001 | | 32.58 (10.92) | | | f(5,3025)=8.18, p<0.001 | | 10.79 (6.48) | | | f(5,3025)=17.60, p<0.001 |
|  | Government Sector | | 8.60 (8.46) | | |  | | | | 4.56 (5.92) | |  | | 7.61 (7.83) | |  | | 29.28 (10.50) | | |  | | 7.55 (6.21) | | |  |
|  | Healthcare | | 9.27 (8.21) | | |  | | | | 4.20 (5.21) | |  | | 7.28 (7.60) | |  | | 28.50 (10.30) | | |  | | 7.35 (6.03) | | |  |
|  | Freelance/Private Sector | | 7.67 (8.45) | | |  | | | | 3.90 (5.27) | |  | | 7.10 (7.94) | |  | | 28.24 (10.23) | | |  | | 7.30 (5.99) | | |  |
|  | Unemployed | | 9.24 (8.83) | | |  | | | | 5.16 (6.53) | |  | | 8.36 (8.47) | |  | | 29.42 (10.38) | | |  | | 8.50 (6.04) | | |  |
|  | Trade | | 10.94 (8.85) | | |  | | | | 5.36 (5.96) | |  | | 8.94 (8.17) | |  | | 31.68 (10.88) | | |  | | 9.68 (5.83) | | |  |
|  | **Region of residence in Saudi Arabia** |  | | |  | | |  | | |  | |  | |  | | | |  | | |  | | |  | |
|  | Eastern region | | 8.81 (8.58) | | | f(4,3027)=3.97, p=0.003 | | | | 4.46 (5.69) | | f(4,3027)=1.82, p=0.123 | | 7.32 (7.90) | | f(4,715.90)=5.30, p<0.001 | | 28.83 (10.17) | | | f(4,3027)=1.36, p=0.245 | | 7.94 (6.05) | | | f(4,3029)=1.73, p=0.085 |
|  | Western regions | | 9.39 (8.87) | | |  | | | | 4.85 (6.12) | |  | | 8.34 (8.19) | |  | | 29.75 (10.34) | | |  | | 8.04 (6.23) | | |  |
|  | Northern regions | | 7.45 (7.80) | | |  | | | | 3.86 (5.43) | |  | | 6.60 (7.50) | |  | | 28.73 (9.94) | | |  | | 6.71 (5.46) | | |  |
|  | Southern regions | | 7.50 (7.66) | | |  | | | | 4.20 (5.48) | |  | | 6.73 (7.06) | |  | | 28.24 (10.38) | | |  | | 7.47 (5.92) | | |  |
|  | Central regions | | 9.27 (8.82) | | |  | | | | 4.85 (6.22) | |  | | 8.40 (8.60) | |  | | 29.53 (10.73) | | |  | | 8.29 (6.34) | | |  |
| q6 | **Contact with patients at work:** | | |  | | |  | |  | | | |  | | | |  | | |  | | | |  | | |
|  | Yes | | 9.12 (8.45) | | | t(3030)=0.396, p=0.692 | | | | 4.27 (5.35) | | t(1048.70)=2.10, p=0.037 | | 7.56 (8.13) | | t(3030)=1.52, p=0.129 | | 28.69 (10.70) | | | t(3030)=1.72, p=0.085 | | 7.64 (6.11) | | | t(3029)=1.72, p=0.085 |
|  | No | | 8.97 (8.74) | | |  | | | | 4.79 ( 6.19) | |  | | 8.13 (8.31) | |  | | 29.51 (10.44) | | |  | | 8.13 (6.23) | | |  |
| q7 | **Family member with patient contact at work:** | |  | | |  | | | |  | |  | |  | |  | |  | | |  | |  | | |  |
|  | Yes | | 9.01 (8.45) | | | t(3030)=0.02, p=0.986 | | | | 4.63 (5.63) | | t(2510.01)=0.40, p=0.690 | | 8.01 (8.21) | | t(3030)=0.0001, p=1 | | 29.27 (10.65) | | | t(3030)=0.33, p=0.740 | | 7.97 (6.30) | | | t(3029)=0.38, p=0.704 |
|  | No | | 8.10 (8.81) | | |  | | | | 4.72 (6.26) | |  | | 8.01 (8.31) | |  | | 29.40 (10.42) | | |  | | 8.06 (6.15) | | |  |
| q8 | **Had contact with a COVID-19 suspected or confirmed case:** | |  | | |  | | | |  | |  | |  | |  | |  | | |  | |  | | |  |
|  | Yes | | 10.52 (8.70) | | | f(2,357.7)=36.10, p<0.001 | | | | 5.75 (5.96) | | f(2,357.04)=30.59, p<0.001 | | 8.48 (8.06) | | f(2,357.92)=25.92, p<0.001 | | 30.65 (10.62) | | | f(2,3029)=17.50,p<0.001 | | 8.49 (6.57) | | | f(2,3028)=19.70,p<0.001 |
|  | No | | 8.04 (8.14) | | |  | | | | 4.06 (5.57) | |  | | 7.24 (7.67) | |  | | 28.58 (10.31) | | |  | | 7.56 (6.09) | | |  |
|  | Unsure | | 11.12 (9.53) | | |  | | | | 6.10 (6.86) | |  | | 9.86 (9.40) | |  | | 31.04 (10.74) | | |  | | 9.13 (6.30) | | |  |
|  | **Chronic illness** | |  | | |  | | | |  | |  | |  | |  | |  | | |  | |  | | |  |
|  | Yes | | 9.10 (9.23) | | | t(3030)=0.260, p=.798 | | | | 5.12 (6.28) | | t(3030)=2.10, p=0.036 | | 8.07 (8.75) | | t(3030)=0.20, p=0.839 | | 28.87 (10.79) | | | f(3030)=1.34,p=0.180 | | 8.26 (6.47) | | | t(3029)=1.10, p=0.275 |
|  | No | | 8.98 (8.51) | | |  | | | | 4.56 (5.96) | |  | | 7.99 (8.14) | |  | | 29.49 (10.41) | | |  | | 7.96 (6.13) | | |  |
|  | **Diagnosed with mental illness** | |  | | |  | | | |  | |  | |  | |  | |  | | |  | |  | | |  |
|  | Yes | | 14.31 (10.40) | | | t(743.50)=14.41, p<0.001 | | | | 8.40 (8.22) | | t(689.25)=12.98, p<0.001 | | 13.28 (10.67) | | t(708.52)=17.1, p<0.001 | | 34.87 (11.40) | | | t(801.8)=13.36,p<0.001 | | 11.43 (6.53) | | | t(820.1)=14.30, p<0.001 |
|  | No | | 7.73 (7.70) | | |  | | | | 3.80 (4.99) | |  | | 6.76 (7.04) | |  | | 28.03 (9.83) | | |  | | 7.22 (5.84) | | |  |
|  | **Depressive Disorders** | |  | | |  | | | |  | |  | |  | |  | |  | | |  | |  | | |  |
|  | No | | 8.16 (8.04) | | | t(360.04)=13.04, p<0.001 | | | | 4.17 (5.50) | | t(349.73)=10.50, p<0.001 | | 7.13 (7.41) | | t(350.50)=13.40, p<0.001 | | 28.55 (10.10) | | | t(377.11)=11.5,p<0.001 | | 7.50 (5.94) | | | t(378.33)=13, p<0.001 |
|  | Yes | | 16.15 (10.55) | | |  | | | | 9.12 (8.23) | |  | | 15.59 (10.97) | |  | | 36.19 (11.32) | | |  | | 12.54 (6.60) | | |  |
|  | **Anxiety Disorders** | |  | | |  | | | |  | |  | |  | |  | |  | | |  | |  | | |  |
|  | No | | 8.28 (8.17) | | | t(392.95)=11.04, p<0.001 | | | | 4.10 (5.38) | | t(373.10)=11.24, p<0.001 | | 7.34 (7.70) | | t(385.90)=10.40, p<0.001 | | 28.53 (10.10) | | | t(408.7)=11.40,p<0.001 | | 7.56 (6.00) | | | t(413.4)=11.3, p<0.001 |
|  | Yes | | 14.71 (10.34) | | |  | | | | 9.39 (8.47) | |  | | 13.41 (10.42) | |  | | 35.87 (11.31) | | |  | | 11.76 (6.52) | | |  |
|  | **Neurodevelopmental Disorder** | |  | | |  | | | |  | |  | |  | |  | |  | | |  | |  | | |  |
|  | No | | 8.99 (8.70) | | | t(3030)=0.35, p=0.729 | | | | 4.69 (6.04) | | t(3030)=0.315, p=0.917 | | 8.02 (8.28) | | t(3030)=0.37, p=0.714 | | 29.35 (10.50) | | | t(3030)=0.83, p=0.407 | | 8.03 (6.21) | | | t(3029)=0.98, p=0.329 |
|  | Yes | | 10.50 (5.97) | | |  | | | | 5.00 (8.72) | |  | | 6.50 (4.43) | |  | | 25.00 (6.68) | | |  | | 5.00 (5.29) | | |  |
|  | **Psychotic Disorders** | |  | | |  | | | |  | |  | |  | |  | |  | | |  | |  | | |  |
|  | No | | 8.99 (8.66) | | | t(9.03)=0.53, p=0.609 | | | | 4.68 (6.03) | | t(3030)=1.32, p=0.178 | | 8.01 (8.26) | | t(3030)=0.70, p=0.494 | | 29.33 (10.49) | | | t(3030)=1.86, p=0.063 | | 8.02 (6.20) | | | t(3029)=2.10, p=0.038 |
|  | Yes | | 11.20 (13.18) | | |  | | | | 7.20 (8.23) | |  | | 9.80 (11.56) | |  | | 35.5 (11.27) | | |  | | 12.1 (5.92) | | |  |
|  | **Bipolar Disorders** | |  | | |  | | | |  | |  | |  | |  | |  | | |  | |  | | |  |
|  | No | | 8.96 (8.63) | | | t(21.13)=1.76, p=0.094 | | | | 4.66 (5.99) | | t(21.10)=1.74, p=0.097 | | 7.97 (8.22) | | t(21.1)=2.10, p=0.053 | | 29.31 (10.48) | | | t(3030)=2.45, p=0.014 | | 8.01 (6.200) | | | t(3029)=1.80, p=0.077 |
|  | Yes | | 14.00 (13.44) | | |  | | | | 8.36 (10.01) | |  | | 13.64 (12.93) | |  | | 34.82 (11.57) | | |  | | 10.36 (6.72) | | |  |
|  | **Obsessive Compulsive Disorder** | |  | | |  | | | |  | |  | |  | |  | |  | | |  | |  | | |  |
|  | No | | 8.89 (8.60) | | | t(73.12)=3.35, p=0.001 | | | | 4.61 (5.94) | | t(72.7)=3.43, p=0.001 | | 7.90 (8.12) | | t(72.53)=3.30, p=0.001 | | 29.19 (10.40) | | | t(73.42)=4.41, p<0.001 | | 7.96 (6.18) | | | t(3029)=3.81 p<0.001 |
|  | Yes | | 13.28 (11.01) | | |  | | | | 8.06 (8.49) | |  | | 12.69 (12.26) | |  | | 35.72 (12.47) | | |  | | 10.78 (6.52) | | |  |
|  | **Trauma Related Disorders** | |  | | |  | | | |  | |  | |  | |  | |  | | |  | |  | | |  |
|  | No | | 8.92 (8.63) | | | t(3030)=4.62, p<0.001 | | | | 4.62 (5.98) | | t(35.50)=3.90, p0.001 | | 7.92 (8.21) | | t(35.60)=4.82, p<0.001 | | 29.26 (10.46) | | | t(3030)=4.30, p<0.001 | | 7.97 (6.19) | | | t(36.37)=6.40, p<0.001 |
|  | Yes | | 15.61 (9.95) | | |  | | | | 9.83 (8.05) | |  | | 15.78 (9.75) | |  | | 36.72 (11.68) | | |  | | 13.19 (4.89) | | |  |
|  | **Eating Disorders** | |  | | |  | | | |  | |  | |  | |  | |  | | |  | |  | | |  |
|  | No | | 8.95 (8.64) | | | t(3030)=3.25, p=0.001 | | | | 4.65 (6.03) | | t(3030)=3.74, p<0.001 | | 7.95 (8.22) | | t(27.30)=3.22, p=0.003 | | 29.29 (10.48) | | | t(3030)=3.12, p=0.002 | | 7.98 (6.18) | | | t(3029)=4.55, p<0.001 |
|  | Yes | | 14.29 (10.31) | | |  | | | | 8.93 (5.97) | |  | | 14.57 (10.84) | |  | | 35.5 (11.02) | | |  | | 13.32 (7.17) | | |  |
|  | **Sleep Disorders** | |  | | |  | | | |  | |  | |  | |  | |  | | |  | |  | | |  |
|  | No | | 8.83 (8.60) | | | t(97.89)=4.89, p<0.001 | | | | 4.57 (5.92) | | t(97.10)=4.22,p<0.001 | | 7.87 (8.17) | | t(98.11)=4.56, p<0.001 | | 29.21 (10.45) | | | t(3030)=4.20, p<0.001 | | 7.81 (6.09) | | | t(3029)=11.34, p<0.001 |
|  | Yes | | 14.27 (10.74) | | |  | | | | 8.21 (8.33) | |  | | 12.59 (9.99) | |  | | 33.74 (11.02) | | |  | | 14.99 (5.80) | | |  |
|  | **Personality Disorders** | |  | | |  | | | |  | |  | |  | |  | |  | | |  | |  | | |  |
|  | No | | 8.93 (8.64) | | | t(3030)=4.10, p<0.001 | | | | 4.62 (5.96) | | t(30.24)=3.54, P=0.001 | | 7.94 (8.22) | | t(30.40)=3.82, p=0.001 | | 29.25 (10.44) | | | t(3030)=4.90, p<0.001 | | 7.99 (6.19) | | | t(3029)=3.60, p<0.001 |
|  | Yes | | 15.29 (9.78) | | |  | | | | 10.71 (9.56) | |  | | 15.10 (10.40) | |  | | 38.48 (12.57) | | |  | | 12.10 (6.12) | | |  |
|  | **Other Disorders** | |  | | |  | | | |  | |  | |  | |  | |  | | |  | |  | | |  |
|  | No | | 8.97 (8.65) | | | t(3030)=0.87, p=0.383 | | | | 4.64 (5.96) | | t(82.21)=1.70, p=0.093 | | 7.96 (8.21) | | t(82.93)=1.91, p=0.059 | | 29.27 (10.44) | | | t(83.20)=2.14, p=0.035 | | 7.99 (6.19) | | | t(3029)=2.13, p=0.033 |
|  | Yes | | 9.83 (9.63) | | |  | | | | 6.25 (8.42) | |  | | 10.12 (10.10) | |  | | 32.22 (12.30) | | |  | | 9.48 (6.64) | | |  |

|  | Appendix Table 3: The Bivariate Analysis of Depression, Anxiety, and Stress (DASS-21) subscales, Insomnia Severity (ISI), and Intolerance of Uncertainty (IUS) across different answers to COVID19 knowledge. | | | | | | | | | | | | | | | | | | | | | | | | | | | | | | | | | | | | | | | | | | | | | | | | | | | | | | | | | | | | | | | | | | | | | | | | | | | | | | | | | | | | | | | | | | | | | | | | | | | | | | | | | | |
| --- | --- | --- | --- | --- | --- | --- | --- | --- | --- | --- | --- | --- | --- | --- | --- | --- | --- | --- | --- | --- | --- | --- | --- | --- | --- | --- | --- | --- | --- | --- | --- | --- | --- | --- | --- | --- | --- | --- | --- | --- | --- | --- | --- | --- | --- | --- | --- | --- | --- | --- | --- | --- | --- | --- | --- | --- | --- | --- | --- | --- | --- | --- | --- | --- | --- | --- | --- | --- | --- | --- | --- | --- | --- | --- | --- | --- | --- | --- | --- | --- | --- | --- | --- | --- | --- | --- | --- | --- | --- | --- | --- | --- | --- | --- | --- | --- | --- | --- | --- | --- | --- | --- | --- | --- | --- | --- | --- |
|  |  |  | | | | | | | | | **N** | | | | | | | | | | **Mean (SD)- Stress** | | | | | | | | | **p-value** | | | | | | | | **Mean (SD)-Anxiety** | | | | | | | | | | **p-value** | | | | | | | **Mean (SD)- Depression** | | | | | | | **p-value** | | | | | | | | | | | **Mean (SD) Uncertainty IUS** | | | | | | | **p-value** | | | | | | | | | **Mean (SD)-Insomnia** | | | | | | | | | |  | | | | | | | | **p-value** |
|  | **Is the cause for COVID-19 known?** | | | | |  | | | | | | | | | | |  | | | | | | | | | | |  | | | | | | | | | | | |  | | | | | | | | | | |  | | | | | | | | |  | | | | | | | | | | |  | | | | | | | | | | | |  | | | | | | | | | | |  | | | | | | | | | |  | | | |
|  | Yes |  | | | | | | | | | 1149 | | | | | | | | | | 8.93 (8.42) | | | | | | | | | f(2,3029)=1.59,p=0.203 | | | | | | | | 4.50 (5.84) | | | | | | | | | | f(2,3029)=1.1, p=0.333 | | | | | | | 7.89 (8.18) | | | | | | | f(2,3029)=0.21, p=0.812 | | | | | | | | | | | 29.62 (10.42) | | | | | | | f(2,3029)=1.14, p=0.319 | | | | | | | | | 7.92 (6.22) | | | | | | | | | | 6.21875 | | | | | | | | f(2,3028)=0.47, p=0.626 |
|  | No |  | | | | | | | | | 970 | | | | | | | | | | 8.98 (8.82) | | | | | | | | |  | | | | | | | | 4.72 (6.13) | | | | | | | | | |  | | | | | | | 8.11 (8.43) | | | | | | |  | | | | | | | | | | | 29.43 (10.76) | | | | | | |  | | | | | | | | | 8.18 (6.19) | | | | | | | | | | 6.1887 | | | | | | | |  |
|  | I don't know |  | | | | | | | | | 913 | | | | | | | | | | 9.10 (8.86) | | | | | | | | |  | | | | | | | | 4.89 (6.18) | | | | | | | | | |  | | | | | | | 8.07 (8.23) | | | | | | |  | | | | | | | | | | | 28.93 (10.32) | | | | | | |  | | | | | | | | | 8.00 (6.21) | | | | | | | | | | 6.21284 | | | | | | | |  |
|  | **Can COVID-19 be transmitted between people?** | | | | | | |  | | | | | | | | | | |  | | | | | | | | | | | | |  | | | | | | | | | | | | |  | | | | | | | | | |  | | | | | | | | | |  | | | | | | | | | | | | |  | | | | | | | | | | | | |  | | | | | | | | | | |  | | | | | |
|  | Yes |  | | | | | | | | | 2981 | | | | | | | | | | 9.03 (8.68) | | | | | | | | | f(2,3029)=1.65,p=0.193 | | | | | | | | 4.69 (6.04) | | | | | | | | | | f(2,16.14)=1.60, p=0.240 | | | | | | | 8.03 (8.28) | | | | | | | f(2,3029)=1.03, p=0.357 | | | | | | | | | | | 29.38 (10.49) | | | | | | | f(2,3029)=1.50, p=0.224 | | | | | | | | | 8.04 (6.21) | | | | | | | | | | 6.20898 | | | | | | | | f(2,3028)=0.17, p=0.845 |
|  | No |  | | | | | | | | | 8 | | | | | | | | | | 10.5 (12.91) | | | | | | | | |  | | | | | | | | 8.50 (9.10) | | | | | | | | | |  | | | | | | | 10.5 (11.55) | | | | | | |  | | | | | | | | | | | 31.75 (11.41) | | | | | | |  | | | | | | | | | 7.88 (5.62) | | | | | | | | | | 5.61726 | | | | | | | |  |
|  | I don't know |  | | | | | | | | | 43 | | | | | | | | | | 6.70 (7.33) | | | | | | | | |  | | | | | | | | 3.72 (4.65) | | | | | | | | | |  | | | | | | | 6.56 (7.06) | | | | | | |  | | | | | | | | | | | 26.79 (11.22) | | | | | | |  | | | | | | | | | 7.49 (6.23) | | | | | | | | | | 6.23115 | | | | | | | |  |
|  | **Can a person have COVID-19 without having had contact with someone carrying the virus?** | | | | | | | | | | | | | |  | | | | | | | | | | | | | | | | | | | | |  | | | | | | | | | | | | | | | | | | |  | | | | | | | | | | | | | | | | | | | |  | | | | | | | | | | | | | | | | | | | | |  | | | | | | | | | | | |
|  | Yes |  | | | | | | | | | 1268 | | | | | | | | | | 9.73 (9.17) | | | | | | | | | f(2,1413.11)=11.57,p<0.001 | | | | | | | | 5.10 (6.47) | | | | | | | | | | f(2,1393.5)=11.30, p<0.001 | | | | | | | 8.56 (8.68) | | | | | | | f(2,1404.7)=8.5, p<0.001 | | | | | | | | | | | 30.04 (10.85) | | | | | | | f(2,1439.3)=5.5, p=0.004 | | | | | | | | | 8.52 (6.36) | | | | | | | | | | 6.36179 | | | | | | | | f(2,1457.04)=9.15, p<0.001 |
|  | No |  | | | | | | | | | 1240 | | | | | | | | | | 8.12 (7.97) | | | | | | | | |  | | | | | | | | 4.08 (5.36) | | | | | | | | | |  | | | | | | | 7.29 (7.62) | | | | | | |  | | | | | | | | | | | 28.65 (10.13) | | | | | | |  | | | | | | | | | 7.47 (6.11) | | | | | | | | | | 6.10922 | | | | | | | |  |
|  | I don't know |  | | | | | | | | | 524 | | | | | | | | | | 9.31 (8.88) | | | | | | | | |  | | | | | | | | 5.12 (6.33) | | | | | | | | | |  | | | | | | | 8.41 (8.62) | | | | | | |  | | | | | | | | | | | 29.32 (10.40) | | | | | | |  | | | | | | | | | 8.18 (5.95) | | | | | | | | | | 5.95016 | | | | | | | |  |
|  | **Can a person be carrying the virus causing COVID-19 without having any symptoms?** | | | | | | | | | | | | | |  | | | | | | | | | | | | | | | | | | | | |  | | | | | | | | | | | | | | | | | | |  | | | | | | | | | | | | | | | | | | | |  | | | | | | | | | | | | | | | | | | | | |  | | | | | | | | | | | |
|  | Yes |  | | | | | | | | | 2764 | | | | | | | | | | 9.17 (8.78) | | | | | | | | | f(2,147.15)=12.42,p<0.001 | | | | | | | | 4.76 (6.10) | | | | | | | | | | f(2,146.51)=5.82, p=0.004 | | | | | | | 8.17 (8.38) | | | | | | | f(2,150.30)=12.5, p<0.001 | | | | | | | | | | | 29.56 (10.50) | | | | | | | f(2,3029)=6.90, p=0.001 | | | | | | | | | 8.13 (6.23) | | | | | | | | | | 6.23163 | | | | | | | | f(2,144.74)=7.55, p=0.001 |
|  | No |  | | | | | | | | | 67 | | | | | | | | | | 5.76 (6.26) | | | | | | | | |  | | | | | | | | 2.99 (4.41) | | | | | | | | | |  | | | | | | | 5.22 (5.18) | | | | | | |  | | | | | | | | | | | 26.27 (8.66) | | | | | | |  | | | | | | | | | 5.94 (4.82) | | | | | | | | | | 4.82066 | | | | | | | |  |
|  | I don't know |  | | | | | | | | | 201 | | | | | | | | | | 7.64 (7.66) | | | | | | | | |  | | | | | | | | 4.22 (5.56) | | | | | | | | | |  | | | | | | | 6.78 (7.28) | | | | | | |  | | | | | | | | | | | 27.43 (10.68) | | | | | | |  | | | | | | | | | 7.42 (6.13) | | | | | | | | | | 6.13388 | | | | | | | |  |
|  | **Are there cases of COVID-19 in Saudi Arabia** | | | | | | |  | | | | | | | | | | |  | | | | | | | | | | | | |  | | | | | | | | | | | | |  | | | | | | | | | |  | | | | | | | | | |  | | | | | | | | | | | | |  | | | | | | | | | | | | |  | | | | | | | | | | |  | | | | | |
|  | Yes |  | | | | | | | | | 2987 | | | | | | | | | | 9.02 (8.69) | | | | | | | | | f(2,3029)=0.885,p=0.413 | | | | | | | | 4.69 (6.03) | | | | | | | | | | f(2,3029)=0.43, p=0.652 | | | | | | | 8.02 (8.26) | | | | | | | f(2,3029)=0.24, p=0.788 | | | | | | | | | | | 29.40 (10.51) | | | | | | | f(2,2029)=2.75, p=0.064 | | | | | | | | | 8.05 (6.21) | | | | | | | | | | 6.21347 | | | | | | | | f(2,3028)=0.90, p=0.428 |
|  | No |  | | | | | | | | | 12 | | | | | | | | | | 7.33 (11.70) | | | | | | | | |  | | | | | | | | 6.00 (9.30) | | | | | | | | | |  | | | | | | | 8.17 (13.71) | | | | | | |  | | | | | | | | | | | 27.33 (11.18) | | | | | | |  | | | | | | | | | 7.83 (6.78) | | | | | | | | | | 6.7801 | | | | | | | |  |
|  | I don't know |  | | | | | | | | | 33 | | | | | | | | | | 7.27 (6.32) | | | | | | | | |  | | | | | | | | 4.12 (5.22) | | | | | | | | | |  | | | | | | | 7.03 (6.73) | | | | | | |  | | | | | | | | | | | 25.27 (8.71) | | | | | | |  | | | | | | | | | 6.64 (5.29) | | | | | | | | | | 5.29043 | | | | | | | |  |
|  | **Is there a treatment for COVID-19?** | | | | | | | | |  | | | | | | | | | | | | | | |  | | | | | | | | | | | | | | | | |  | | | | | | | | | | | | |  | | | | | | | | | | | | | |  | | | | | | | | | | | | | | | |  | | | | | | | | | | | | | | | |  | | | | | | |
|  | Yes |  | | | | | | | | | 287 | | | | | | | | | | 8.15 (8.48) | | | | | | | | | f(2,3029)=8.56,p<0.001 | | | | | | | | 3.92 (5.70) | | | | | | | | | | f(2,795.62)=3.39, p=0.034 | | | | | | | 7.00 (7.60) | | | | | | | f(2,804.42)=5.18, p=0.006 | | | | | | | | | | | 28.44 (10.15) | | | | | | | f(2,790.8)=6.18, p=0.002 | | | | | | | | | 7.51 (6.12) | | | | | | | | | | 6.12336 | | | | | | | | f(2,3028)=1.23, p=0.293 |
|  | No |  | | | | | | | | | 1765 | | | | | | | | | | 9.55 (8.88) | | | | | | | | |  | | | | | | | | 4.86 (6.18) | | | | | | | | | |  | | | | | | | 8.38 (8.55) | | | | | | |  | | | | | | | | | | | 29.91 (10.81) | | | | | | |  | | | | | | | | | 8.13 (6.27) | | | | | | | | | | 6.26703 | | | | | | | |  |
|  | I don't know |  | | | | | | | | | 980 | | | | | | | | | | 8.25 (8.30) | | | | | | | | |  | | | | | | | | 4.60 (5.86) | | | | | | | | | |  | | | | | | | 7.65 (7.92) | | | | | | |  | | | | | | | | | | | 28.61 (9.96) | | | | | | |  | | | | | | | | | 8.01 (6.12) | | | | | | | | | | 6.11834 | | | | | | | |  |
|  | **Is COVID-19 considered deadlier than previous respiratory infection epidemics (such as MERS, Swine Flu, or Bird Flu)?** | | | | | | | | | | | | | | | | | | | | | | | | | | | | | | | | | | |  | | | | | | | | | | | | | | | | | | | | | | | | | | | | | | | | | | | | | | |  | | | | | | | | | | | | | | | | | | | | | | | | | | | | | | | | |
|  | Yes |  | | | | | | | | | 880 | | | | | | | | | | 9.20 (8.56) | | | | | | | | | f(2,3029)=0.365,p=0.694 | | | | | | | | 4.79 (6.10) | | | | | | | | | | f(2,3029)=0.42, p=0.656 | | | | | | | 8.11 (8.29) | | | | | | | f(2,3029)=0.87, p=0.421 | | | | | | | | | | | 29.57 (10.68) | | | | | | | f(2,2029)=1.96, p=0.141 | | | | | | | | | 7.95 (6.19) | | | | | | | | | | 6.18613 | | | | | | | | f(2,3028)=0.60, p=0.550 |
|  | No |  | | | | | | | | | 1379 | | | | | | | | | | 8.94 (8.91) | | | | | | | | |  | | | | | | | | 4.58 (5.60) | | | | | | | | | |  | | | | | | | 7.81 (8.31) | | | | | | |  | | | | | | | | | | | 29.57 (10.68) | | | | | | |  | | | | | | | | | 7.96 (6.25) | | | | | | | | | | 6.24803 | | | | | | | |  |
|  | I don't know |  | | | | | | | | | 773 | | | | | | | | | | 8.86 (8.39) | | | | | | | | |  | | | | | | | | 4.76 (6.13) | | | | | | | | | |  | | | | | | | 8.27 (8.21) | | | | | | |  | | | | | | | | | | | 28.70 (9.95) | | | | | | |  | | | | | | | | | 8.24 (6.16) | | | | | | | | | | 6.15738 | | | | | | | |  |
|  | **Is COVID-19 considered more infectious than previous respiratory infection epidemics (such as MERS, Swine Flu, or Bird Flu)?** | | | | | | | | | | | | | | | | | | | | | | | | | | | | | | | | | | |  | | | | | | | | | | | | | | | | | | | | | | | | | | | | | | | | | | | | | | |  | | | | | | | | | | | | | | | | | | | | | | | | | | | | | | | | |
|  | Yes |  | | | | | | | | | 2388 | | | | | | | | | | 9.18 (8.65) | | | | | | | | | f(2,3029)=2.978,p=0.051 | | | | | | | | 4.71 (6.10) | | | | | | | | | | f(2,3029)=0.96, p=0.383 | | | | | | | 8.09 (8.22) | | | | | | | f(2,3029)=1.35, p=0.259 | | | | | | | | | | | 29.66 (10.50) | | | | | | | f(2,404.4)=6.15, p=0.002 | | | | | | | | | 8.05 (6.14) | | | | | | | | | | 6.14307 | | | | | | | | f(2,3028)=0.50, p=0.613 |
|  | No |  | | | | | | | | | 179 | | | | | | | | | | 7.83 (8.60) | | | | | | | | |  | | | | | | | | 4.10 (5.33) | | | | | | | | | |  | | | | | | | 7.04 (8.36) | | | | | | |  | | | | | | | | | | | 28.92 (11.60) | | | | | | |  | | | | | | | | | 7.59 (6.39) | | | | | | | | | | 6.38617 | | | | | | | |  |
|  | I don't know |  | | | | | | | | | 465 | | | | | | | | | | 8.49 (8.83) | | | | | | | | |  | | | | | | | | 4.79 (6.23) | | | | | | | | | |  | | | | | | | 7.99 (8.52) | | | | | | |  | | | | | | | | | | | 27.89 (9.96) | | | | | | |  | | | | | | | | | 8.08 (6.46) | | | | | | | | | | 6.46192 | | | | | | | |  |
|  | **What are the symptoms of having COVID-19? [Choose the correct answer/s]** | | | | | | | | | | | | | | | | | | | | | | | | | | | | | | | | | | |  | | | | | | | | | | | | | | | | | | | | | | | | | | | | | | | | | | | | | | |  | | | | | | | | | | | | | | | | | | | | | | | | | | | | | | | | |
|  | Fever | | |  | | | | | | | | |  | | | | | | | | | |  | | | | | | | | | | |  | | | | | | | | |  | | | | | | | | | |  | | | | |  | | | | | | | | |  | | | | | | | | |  | | | | | | | | | | |  | | | | | | | | | |  | | | | | | | | |  | |
|  | No |  | | | | | | | | | 139 | | | | | | | | | | 7.96 (7.44) | | | | | | | | | t(156.8)=1.67,p=0.096 | | | | | | | | 4.01 (4.97) | | | | | | | | | | t( 158.6)=1.62, p=0.108 | | | | | | | 7.35 (6.78) | | | | | | | t(158.7)=1.17, p=0.246 | | | | | | | | | | | 28.88 (9.60) | | | | | | | t(3030)=0.53, p=0.594 | | | | | | | | | 7.70 (5.72 ) | | | | | | | | | | 5.71528 | | | | | | | | t(3029)=0.66, p=0.509 |
|  | Yes |  | | | | | | | | | 2893 | | | | | | | | | | 9.05 (8.73) | | | | | | | | |  | | | | | | | | 4.72 (6.10) | | | | | | | | | |  | | | | | | | 8.05 (8.34) | | | | | | |  | | | | | | | | | | | 29.37 (10.50) | | | | | | |  | | | | | | | | | 8.05 (6.23) | | | | | | | | | | 6.22935 | | | | | | | |  |
|  | Cough | | | |  | | | | | | | | | |  | | | | | | | | | |  | | | | | | | | | | |  | | | | | | | | | | |  | | | | | | | |  | | | | | | | | |  | | | | | | | | | | |  | | | | | | | | | |  | | | | | | | | | | |  | | | | | | | | |  | | |
|  | No |  | | | | | | | | | 405 | | | | | | | | | | 8.78 (9.06) | | | | | | | | | t(3030)=0.54,p=0.589 | | | | | | | | 4.73 (6.31) | | | | | | | | | | t(3030)=0.14, p=0.888 | | | | | | | 7.94 (8.57) | | | | | | | t(3030)=0.21, p=0.837 | | | | | | | | | | | 29.42 (10.56) | | | | | | | t(3030)=0.15, p=0.880 | | | | | | | | | 8.06 (6.09) | | | | | | | | | | 6.0949 | | | | | | | | t(3029)=0.10, p=0.927 |
|  | Yes |  | | | | | | | | | 2627 | | | | | | | | | | 9.03 (8.62) | | | | | | | | |  | | | | | | | | 4.68 (5.99) | | | | | | | | | |  | | | | | | | 8.03 (8.23) | | | | | | |  | | | | | | | | | | | 29.34 (10.49) | | | | | | |  | | | | | | | | | 8.03 (6.22) | | | | | | | | | | 6.22441 | | | | | | | |  |
|  | Sneezing | | | |  | | | | | | | | | |  | | | | | | | | | |  | | | | | | | | | | |  | | | | | | | | | | |  | | | | | | | |  | | | | | | | | |  | | | | | | | | | | |  | | | | | | | | | |  | | | | | | | | | | |  | | | | | | | | |  | | |
|  | No |  | | | | | | | | | 1877 | | | | | | | | | | 9.26 (8.82) | | | | | | | | | t(3030)=2.14,p=0.033 | | | | | | | | 4.85 (6.17) | | | | | | | | | | t(3030)=1.95, p=0.051 | | | | | | | 8.20 (8.41) | | | | | | | t(3030)=1.53, p=0.126 | | | | | | | | | | | 29.53 (10.43) | | | | | | | t(3030)=1.21, p=0.227 | | | | | | | | | 8.19 (6.18) | | | | | | | | | | 6.18013 | | | | | | | | t(3029)=1.80, p=0.075 |
|  | Yes |  | | | | | | | | | 1155 | | | | | | | | | | 8.57 (8.44) | | | | | | | | |  | | | | | | | | 4.41 (5.81) | | | | | | | | | |  | | | | | | | 7.72 (8.04) | | | | | | |  | | | | | | | | | | | 29.06 (10.62) | | | | | | |  | | | | | | | | | 7.77 (6.24) | | | | | | | | | | 6.2428 | | | | | | | |  |
|  | Stuffy Nose | | | | |  | | | | | | | | | | |  | | | | | | | | | | |  | | | | | | | | | | | |  | | | | | | | | | | |  | | | | | | | | |  | | | | | | | | | | |  | | | | | | | | | | | |  | | | | | | | | | | |  | | | | | | | | | |  | | | |
|  | No |  | | | | | | | | | 2240 | | | | | | | | | | 9.10 (8.65) | | | | | | | | | t(3030)=0.933,p=0.351 | | | | | | | | 4.67 (5.90) | | | | | | | | | | t(1296.30)=0.30, p=0.781 | | | | | | | 8.11 (8.23) | | | | | | | t(3030)=1.10, p=0.277 | | | | | | | | | | | 29.36 (10.37) | | | | | | | t(3030)=0.10, p=0.917 | | | | | | | | | 8.16 (6.18) | | | | | | | | | | 6.17916 | | | | | | | | t(3029)=1.94, p=0.052 |
|  | Yes |  | | | | | | | | | 792 | | | | | | | | | | 8.75 (8.77) | | | | | | | | |  | | | | | | | | 4.74 (6.41) | | | | | | | | | |  | | | | | | | 7.74 (8.40) | | | | | | |  | | | | | | | | | | | 29.32 (10.86) | | | | | | |  | | | | | | | | | 7.66 (6.27) | | | | | | | | | | 6.27178 | | | | | | | |  |
|  | Shortness of breath | | | |  | | | | | | | | | |  | | | | | | | | | |  | | | | | | | | | | |  | | | | | | | | | | |  | | | | | | | |  | | | | | | | | |  | | | | | | | | | | |  | | | | | | | | | |  | | | | | | | | | | |  | | | | | | | | |  | | |
|  | No |  | | | | | | | | | 112 | | | | | | | | | | 7.46 (7.85) | | | | | | | | | t(3030)=1.91,p=0.057 | | | | | | | | 4.55 (6.37) | | | | | | | | | | t(3030)=0.24, p=0.812 | | | | | | | 7.39 (7.76) | | | | | | | t(3030)=0.71, p=0.418 | | | | | | | | | | | 28.50 (9.89) | | | | | | | t(3030)=0.87, p=0.383 | | | | | | | | | 6.84 (5.49) | | | | | | | | | | 5.44845 | | | | | | | | t(122.4)=2.34, p=0.021 |
|  | Yes |  | | | | | | | | | 2920 | | | | | | | | | | 9.10 (8.70) | | | | | | | | |  | | | | | | | | 4.69 (6.03) | | | | | | | | | |  | | | | | | | 8.04 (8.29) | | | | | | |  | | | | | | | | | | | 29.38 (10.52) | | | | | | |  | | | | | | | | | 8.08 (6.23) | | | | | | | | | | 6.22981 | | | | | | | |  |
| Skin Discoloration | | |  | | | | | | | | | | | | |  | | | | | | | | | | |  | | | | | | | | | |  | | | | | | | | | | | | |  | | | | | | |  | | | | | | | | | | | | |  | | | | | | | | | | | |  | | | | | | | | | | |  | | | | | | | | | |  | | | | |
|  | No |  | | | | | | | | | | 2923 | | | | | | | | | | 8.95 (8.61) | | | | | | | | | t(3030)=01.499,p=0.134 | | | | | | | | 4.66 (5.99) | | | | | | | | | | t(3030)=1.15, p=0.250 | | | | | | | 7.97 (8.21) | | | | | | | t(113.81)=1.44, p=0.154 | | | | | | | | | | | 29.31 (10.40) | | | | | | | t(3030)=0.99, p=0.320 | | | | | | | | | 8.05 (6.19) | | | | | | | | | | 6.18727 | | | | | | | t(3029)=0.70, p=0.485 |
|  | Yes |  | | | | | | | | | | 109 | | | | | | | | | | 10.22 (10.26) | | | | | | | | |  | | | | | | | | 5.34 (7.09) | | | | | | | | | |  | | | | | | | 9.32 (9.73) | | | | | | |  | | | | | | | | | | | 30.33 (12.99) | | | | | | |  | | | | | | | | | 7.62 (6.72) | | | | | | | | | | 6.71825 | | | | | | |  |
|  | Diarrhea |  | | | | | | | | | |  | | | | | | | | | |  | | | | | | | | |  | | | | | | | |  | | | | | | | | | |  | | | | | | |  | | | | | | |  | | | | | | | | | | |  | | | | | | |  | | | | | | | | |  | | | | | | | | | |  | | | | | | |  |
|  | No |  | | | | | | | | | | 2057 | | | | | | | | | | 8.90 (8.59) | | | | | | | | | t(3030)=0.939,p=0.348 | | | | | | | | 4.59 (6.00) | | | | | | | | | | t(3030)=1.14, p=0.250 | | | | | | | 7.98 (8.23) | | | | | | | t(3030)=0.30, p=0.764 | | | | | | | | | | | 29.48 (10.52) | | | | | | | t(3030)=1.00, p=0.315 | | | | | | | | | 8.11 (6.20) | | | | | | | | | | 6.19655 | | | | | | | t(3029)=1.02,p=0.308 |
|  | Yes |  | | | | | | | | | | 975 | | | | | | | | | | 9.21 (8.85) | | | | | | | | |  | | | | | | | | 4.87 (6.11) | | | | | | | | | |  | | | | | | | 8.08 (8.37) | | | | | | |  | | | | | | | | | | | 29.07 (10.46) | | | | | | |  | | | | | | | | | 7.86 (6.23) | | | | | | | | | | 6.22663 | | | | | | |  |
|  | Red eyes | | |  | | | | | | | | | |  | | | | | | | | | |  | | | | | | | | | | |  | | | | | | | | |  | | | | | | | | | |  | | | | |  | | | | | | | | |  | | | | | | | | |  | | | | | | | | | | |  | | | | | | | | | |  | | | | | | | |  | |
|  | No |  | | | | | | | | | | 2708 | | | | | | | | | | 8.98 (8.63) | | | | | | | | | t(3030)=0.412,p=0.680 | | | | | | | | 4.66 (5.96) | | | | | | | | | | t(3030)=0.70, p=0.499 | | | | | | | 7.96 (8.19) | | | | | | | t(3030)=1.10, p=0.295 | | | | | | | | | | | 29.36 (10.49) | | | | | | | t(3030)=0.15, p=0.880 | | | | | | | | | 8.08 (6.22) | | | | | | | | | | 6.21768 | | | | | | | t(3029)=1.20,p=0.230 |
|  | Yes |  | | | | | | | | | | 324 | | | | | | | | | | 9.19 (9.04) | | | | | | | | |  | | | | | | | | 4.90 (6.34) | | | | | | | | | |  | | | | | | | 8.47 (8.95) | | | | | | |  | | | | | | | | | | | 29.27 (10.63) | | | | | | |  | | | | | | | | | 7.64 (6.11) | | | | | | | | | | 6.10556 | | | | | | |  |
|  | Joint Pains | | |  | | | | | | | | | |  | | | | | | | | | |  | | | | | | | | | | |  | | | | | | | | |  | | | | | | | | | |  | | | | |  | | | | | | | | |  | | | | | | | | |  | | | | | | | | | | |  | | | | | | | | | |  | | | | | | | |  | |
|  | No |  | | | | | | | | | | 1732 | | | | | | | | | | 8.68 (8.44) | | | | | | | | | t(3030)=2.31,p=0.021 | | | | | | | | 4.57 (5.98) | | | | | | | | | | t(3030)=1.22, p=0.221 | | | | | | | 7.76 (8.17) | | | | | | | t(3030)=1.93, p=0.054 | | | | | | | | | | | 29.36 (10.49) | | | | | | | t(3030)=0.50, p=0.624 | | | | | | | | | 7.91 (6.19) | | | | | | | | | | 6.18989 | | | | | | | t(3029)=1.18,p=0.238 |
|  | Yes |  | | | | | | | | | | 1300 | | | | | | | | | | 9.42 (8.97) | | | | | | | | |  | | | | | | | | 4.84 (6.11) | | | | | | | | | |  | | | | | | | 8.35 (8.41) | | | | | | |  | | | | | | | | | | | 29.27 (10.63) | | | | | | |  | | | | | | | | | 8.18 (6.23) | | | | | | | | | | 6.2271 | | | | | | |  |
|  | Anosmia ( loss of sense of smell) | | | | | |  | | | | | | | | | | |  | | | | | | | | | | |  | | | | | | | | | | | |  | | | | | | | | | | |  | | | | | | | | |  | | | | | | | | | | |  | | | | | | | | | | | |  | | | | | | | | | | |  | | | | | | | | |  | | | |
|  | No |  | | | | | | | | | | 1987 | | | | | | | | | | 8.47 (8.32) | | | | | | | | | t(1935.8)=4.44,p<0.001 | | | | | | | | 4.33 (5.72) | | | | | | | | | | t(1892.1)=4.33, p<0.001 | | | | | | | 7.50 (7.87) | | | | | | | t(1906.9)=4.60, p<0.001 | | | | | | | | | | | 28.89 (10.38) | | | | | | | t(3030)=3.32, p=0.001 | | | | | | | | | 7.78 (6.16) | | | | | | | | | | 6.15991 | | | | | | | t(3029)=0.70, p=0.485 |
|  | Yes |  | | | | | | | | | | 1045 | | | | | | | | | | 9.99 (9.25) | | | | | | | | |  | | | | | | | | 5.37 (6.54) | | | | | | | | | |  | | | | | | | 8.99 (8.91) | | | | | | |  | | | | | | | | | | | 30.22 (10.68) | | | | | | |  | | | | | | | | | 8.50 (6.27) | | | | | | | | | | 6.26913 | | | | | | |  |
|  | **What are the recommended methods for preventing the spread of COVID-19? [Choose the correct answer/s]** | | | | | | | | | | | | | | | | | | | | | | | | |  | | | | | | | | | | | | | | | | | | | | | | | | | | | | | |  | | | | | | | | | | | | | | | | | | | | | | | | | | | | | |  | | | | | | | | | | | | | | | | | | | | | |
|  | Wearing medical gloves | | | | | |  | | | | | | | | | | |  | | | | | | | | | | |  | | | | | | | | | | | |  | | | | | | | | | | |  | | | | | | | | |  | | | | | | | | | | |  | | | | | | | | | | | |  | | | | | | | | | | |  | | | | | | | | |  | | | |
|  | No |  | | | | | | | | | | 1470 | | | | | | | | | | 9.65 (8.91) | | | | | | | | | t(2988.4)=3.99,p<0.001 | | | | | | | | 5.08 (6.24) | | | | | | | | | | t(2978.4)=3.50, p=0.001 | | | | | | | 8.53 (8.48) | | | | | | | t(3030)=3.36, p=0.001 | | | | | | | | | | | 29.77 (10.64) | | | | | | | t(3006.3)=2.20, p=0.031 | | | | | | | | | 8.19 (6.16) | | | | | | | | | | 6.157 | | | | | | | t(3029)=1.41, p=0.159 |
|  | Yes |  | | | | | | | | | | 1562 | | | | | | | | | | 8.39 (8.41) | | | | | | | | |  | | | | | | | | 4.32 (5.82) | | | | | | | | | |  | | | | | | | 7.53 (8.05) | | | | | | |  | | | | | | | | | | | 28.95 (10.35) | | | | | | |  | | | | | | | | | 7.88 (6.25) | | | | | | | | | | 6.25039 | | | | | | |  |
|  | Not touching one’s face | | | | | |  | | | | | | | | | | |  | | | | | | | | | | |  | | | | | | | | | | | |  | | | | | | | | | | |  | | | | | | | | |  | | | | | | | | | | |  | | | | | | | | | | | |  | | | | | | | | | | |  | | | | | | | | |  | | | |
|  | No |  | | | | | | | | | | 411 | | | | | | | | | | 7.75 (7.92) | | | | | | | | | t(579.87)=3.39,p=0.001 | | | | | | | | 4.13 (5.53) | | | | | | | | | | t(579.10)=2.20, p=0.031 | | | | | | | 7.33 (7.81) | | | | | | | t(3030)=1.81, p=0.071 | | | | | | | | | | | 27.75 (9.71) | | | | | | | t(574.20)=3.53, p<0.001 | | | | | | | | | 7.09 (5.66) | | | | | | | | | | 5.66074 | | | | | | | t(579.96)=3.60, p<0.001 |
|  | Yes |  | | | | | | | | | | 2621 | | | | | | | | | | 9.19 (8.78) | | | | | | | | |  | | | | | | | | 4.78 (6.11) | | | | | | | | | |  | | | | | | | 8.12 (8.34) | | | | | | |  | | | | | | | | | | | 29.60 (10.60) | | | | | | |  | | | | | | | | | 8.18 (6.28) | | | | | | | | | | 6.27566 | | | | | | |  |
|  | Staying at home & social distancing | | | | | |  | | | | | | | | | | |  | | | | | | | | | | |  | | | | | | | | | | | |  | | | | | | | | | | |  | | | | | | | | |  | | | | | | | | | | |  | | | | | | | | | | | |  | | | | | | | | | | |  | | | | | | | | |  | | | |
|  | No |  | | | | | | | | | | 64 | | | | | | | | | | 8.25 (8.14) | | | | | | | | | t(3030)=0.70, p=0.486 | | | | | | | | 4.25 (5.91) | | | | | | | | | | t(3030)=0.60, p=0.559 | | | | | | | 7.47 (8.34) | | | | | | | t(3030)=0.53, p=0.594 | | | | | | | | | | | 28.03 (10.34) | | | | | | | t(3030)=1.02, p=0.310 | | | | | | | | | 6.84 (5.23) | | | | | | | | | | 5.22879 | | | | | | | t(3029)=1.55, p=0.122 |
|  | Yes |  | | | | | | | | | | 2968 | | | | | | | | | | 9.01 (8.69) | | | | | | | | |  | | | | | | | | 4.70 (6.04) | | | | | | | | | |  | | | | | | | 8.03 (8.27) | | | | | | |  | | | | | | | | | | | 29.38 (10.50) | | | | | | |  | | | | | | | | | 8.06 (6.22) | | | | | | | | | | 6.22393 | | | | | | |  |
|  | Washing Hands with water and soap | | | | | |  | | | | | | | | | | |  | | | | | | | | | | |  | | | | | | | | | | | |  | | | | | | | | | | |  | | | | | | | | |  | | | | | | | | | | |  | | | | | | | | | | | |  | | | | | | | | | | |  | | | | | | | | |  | | | |
|  | No |  | | | | | | | | | | 93 | | | | | | | | | | 9.20 (9.18) | | | | | | | | | t(3030)=0.23, p= 0.815 | | | | | | | | 5.01 (6.60) | | | | | | | | | | t(3030)=0.53, p=0.599 | | | | | | | 7.48 (8.19) | | | | | | | t(3030)=0.63, p=0.530 | | | | | | | | | | | 28.28 (10.60) | | | | | | | t(3030)=1.00, p=0.319 | | | | | | | | | 8.31 (6.17) | | | | | | | | | | 6.17495 | | | | | | | t(3029)=0.44, p=0.657 |
|  | Yes |  | | | | | | | | | | 2939 | | | | | | | | | | 8.99 (8.88) | | | | | | | | |  | | | | | | | | 4.68 (6.02) | | | | | | | | | |  | | | | | | | 8.03 (8.28) | | | | | | |  | | | | | | | | | | | 29.38 (10.50) | | | | | | |  | | | | | | | | | 8.02 (6.21) | | | | | | | | | | 6.20811 | | | | | | |  |
|  | Not shaking hands | | | | | |  | | | | | | | | | | |  | | | | | | | | | | |  | | | | | | | | | | | |  | | | | | | | | | | |  | | | | | | | | |  | | | | | | | | | | |  | | | | | | | | | | | |  | | | | | | | | | | |  | | | | | | | | |  | | | |
|  | No |  | | | | | | | | | | 177 | | | | | | | | | | 8.80 (9.13) | | | | | | | | | t(3030)=0.31, p= 0.758 | | | | | | | | 4.75 (6.28) | | | | | | | | | | t(3030)=0.13, p=0.893 | | | | | | | 7.82 (9.03) | | | | | | | t(3030)=0.32, p=0.746 | | | | | | | | | | | 28.47 (10.11) | | | | | | | t(3030)=1.15, p=0.251 | | | | | | | | | 7.59 (5.98) | | | | | | | | | | 5.97955 | | | | | | | t(3029)=0.97, p=0.328 |
|  | Yes |  | | | | | | | | | | 2855 | | | | | | | | | | 9.00 (8.65) | | | | | | | | |  | | | | | | | | 4.68 (6.02) | | | | | | | | | |  | | | | | | | 8.03 (8.23) | | | | | | |  | | | | | | | | | | | 29.40 (10.52) | | | | | | |  | | | | | | | | | 8.06 (6.22) | | | | | | | | | | 6.22004 | | | | | | |  |
|  | Wearing medical face masks | | | | | |  | | | | | | | | | | |  | | | | | | | | | | |  | | | | | | | | | | | |  | | | | | | | | | | |  | | | | | | | | |  | | | | | | | | | | |  | | | | | | | | | | | |  | | | | | | | | | | |  | | | | | | | | |  | | | |
|  | No |  | | | | | | | | | | 1749 | | | | | | | | | | 9.38 (8.72) | | | | | | | | | t(3030)=2.80, p=0.005 | | | | | | | | 4.93 (6.13) | | | | | | | | | | t(3030)=2.60, p=0.010 | | | | | | | 8.31 (8.29) | | | | | | | t(3030)=2.32, p=0.020 | | | | | | | | | | | 29.57 (10.56) | | | | | | | t(3030)=1.35, p=0.176 | | | | | | | | | 8.16 (6.15) | | | | | | | | | | 6.15186 | | | | | | | t(3029)=1.30, p=0.195 |
|  | Yes |  | | | | | | | | | | 1283 | | | | | | | | | | 8.48 (8.59) | | | | | | | | |  | | | | | | | | 4.36 (5.89) | | | | | | | | | |  | | | | | | | 7.61 (8.24) | | | | | | |  | | | | | | | | | | | 29.05 (10.42) | | | | | | |  | | | | | | | | | 7.86 (6.28) | | | | | | | | | | 6.27814 | | | | | | |  |
|  | Using tissue when sneezing | | | | | |  | | | | | | | | | | |  | | | | | | | | | | |  | | | | | | | | | | | |  | | | | | | | | | | |  | | | | | | | | |  | | | | | | | | | | |  | | | | | | | | | | | |  | | | | | | | | | | |  | | | | | | | | |  | | | |
|  | No |  | | | | | | | | | | 438 | | | | | | | | | | 9.98 (9.10) | | | | | | | | | t(3030)=2.56, p=0.011 | | | | | | | | 5.37 (6.42) | | | | | | | | | | t(3030)=2.55, p=0.011 | | | | | | | 9.07 (8.58) | | | | | | | t(3030)=2.89, p=0.004 | | | | | | | | | | | 29.89 (10.59) | | | | | | | t(3030)=1.17, p=0.241 | | | | | | | | | 8.84 (6.38) | | | | | | | | | | 6.38054 | | | | | | | t(3029)=2.96, p=0.003 |
|  | Yes |  | | | | | | | | | | 2594 | | | | | | | | | | 8.83 (8.61) | | | | | | | | |  | | | | | | | | 4.57 (5.97) | | | | | | | | | |  | | | | | | | 7.84 (8.21) | | | | | | |  | | | | | | | | | | | 29.26 (10.48) | | | | | | |  | | | | | | | | | 7.89 (6.17) | | | | | | | | | | 6.16712 | | | | | | |  |
|  | Making social gathering shorter | | | | | |  | | | | | | | | | | |  | | | | | | | | | | |  | | | | | | | | | | | |  | | | | | | | | | | |  | | | | | | | | |  | | | | | | | | | | |  | | | | | | | | | | | |  | | | | | | | | | | |  | | | | | | | | |  | | | |
|  | No |  | | | | | | | | | | 2608 | | | | | | | | | | 9.10 (8.71) | | | | | | | | | t(3030)=0.80, p=0.423 | | | | | | | | 4.66 (6.00) | | | | | | | | | | t(3030)=0.53, p=0.598 | | | | | | | 8.06 (8.29) | | | | | | | t(3030)=0.70, p=0.494 | | | | | | | | | | | 29.31 (10.54) | | | | | | | t(3030)=0.50, p=0.618 | | | | | | | | | 8.05 (6.200) | | | | | | | | | | 6.20052 | | | | | | | t(3029)=0.43, p=0.568 |
|  | Yes |  | | | | | | | | | | 424 | | | | | | | | | | 8.68 (8.47) | | | | | | | | |  | | | | | | | | 4.83 (6.27) | | | | | | | | | |  | | | | | | | 7.76 (8.19) | | | | | | |  | | | | | | | | | | | 29.58 (10.27) | | | | | | |  | | | | | | | | | 7.91 (6.25) | | | | | | | | | | 6.24757 | | | | | | |  |
|  | Avoiding close contact with someone with symptoms | | | | | | | |  | | | | | | | | | | |  | | | | | | | | | | | | |  | | | | | | | | | | | | |  | | | | | | | | | |  | | | | | | | | | |  | | | | | | | | | | | | |  | | | | | | | | | | | | |  | | | | | | | | | |  | | | | | |
|  | No |  | | | | | | | | | | 641 | | | | | | | | | | 9.05 (8.22) | | | | | | | | | t(3030)=0.178, p=0.859 | | | | | | | | 4.68 (5.96) | | | | | | | | | | t(3030)=0.10, p=0.964 | | | | | | | 8.55 (8.18) | | | | | | | t(3030)=1.84, p=0.065 | | | | | | | | | | | 29.84 (10.55) | | | | | | | t(3030)=1.33, p=0.148 | | | | | | | | | 8.42 (6.38) | | | | | | | | | | 6.38159 | | | | | | | t(3029)=1.78, p=0.076 |
|  | Yes |  | | | | | | | | | | 2391 | | | | | | | | | | 8.98 (8.80) | | | | | | | | |  | | | | | | | | 4.69 (6.06) | | | | | | | | | |  | | | | | | | 7.87 (8.29) | | | | | | |  | | | | | | | | | | | 29.22 (10.48) | | | | | | |  | | | | | | | | | 7.93 (6.16) | | | | | | | | | | 6.15565 | | | | | | |  |
