# Supplementary material for: Mental health and its association with coping strategies and intolerance of uncertainty during the COVID-19 pandemic among the general population in Saudi Arabia: cross-sectional study
Source: BMC Psychiatry. 2021 Jul 28;21:382. doi: 10.1186/s12888-021-03370-4 (PMC8317145; doi:10.1186/s12888-021-03370-4)
Supplement: Supplementary file 2 — Additional file 2. [file 12888_2021_3370_MOESM2_ESM.zip › Study Questionnaire Arabic FinalR5.pdf]

Study Questionnaire:

Mental Health of the general population in Saudi Arabia during the COVID-19 Pandemic.

Part 0: Introduction and consent:

أعلنت منظمة الصحة العالمية (WHO) في شهر مارس 2020 أن مرض فيروس كورونا 2019 (COVID-19) جائحة عالمية. أدى ذلك إلى جهود دولية للحد من انتشار المرض و تقليل ضرره على البشر، بما في ذلك جهود المملكة العربية السعودية بإيقاف المدارس و منع التجمعات و تفعيل الخدمات الصحية و حضر التجول.

إلّا أن أضرار الأوبئة تتعدى المصابين بها و قد تصل إلى عبء نفسي ينتشر في المجتمع بأكمل. و قد أظهرت دراسات سابقة على أوبئة ماضية انتشار الآثار النفسية للأوبئة في عامة الناس.

نحن فريق من الباحثين في الصحة النفسية، و هدفنا هو تقدير الأثر النفسي للجائحة الحالية على عامة الناس ممّن يسكنون في المملكة العربية السعودية، و دراسة العوامل المؤثرة في ظهور الأعراض النفسية خلال هذه الأزمة.

سيتم سؤالك خلال هذا الاستبيان عن بعض بياناتك، بدون أي دلالات على هويتك الشخصية .
كما سيتم سؤالك عن بعض المعلومات عن جائحة مرض فيروس كورونا 2019 (COVID-19) ، و من ثم تقدير وجود أي أعراض نفسية خلال هذه الفترة و كيفية تكيفك مع الوضع و مقدار تحملك للشك و الغموض.
سيستغرق هذا الاستبيان حوالي 10 دقائق.

نؤكد لك أن جميع إجاباتك التي ستقدمها ستبقى في سرّية تامة مع العلم أنه لا يوجد هناك معلومات يمكن أن تدلنا على هويتك من خلال هذا الاستبيان، ويمكنك التواصل مع د. أحمد الهادي للإجابة على أسئلتك على رقم جوال: 05553380800
وإيميل alhadi@ksu.edu.sa :
إذا كنت موافقاً على المشاركة في هذا الاستبيان الإلكتروني فيرجى النقر على "التالي" للبدء.

Part 1: Demographics:

1.1 العمر:

1.2 الجنس:

1.3 الحالة الاجتماعية:

1.4 المنطقة:

1.5 المهنة\مجال العمل:

1.6 هل مكان عملك يقدم خدمات صحية؟

1.7 هل عملك يتضمن التعامل مع مرضى؟

1.8 هل يعمل أحد أفراد أسرتك في المجال الصحي؟

1.9 هل خالطت شخصاً يحتمل أنه مصاب أو تأكد أنه مصاب بمرض فيروس كورونا 2019 (COVID-19)؟

1.10 هل تعاني من أمراض صحية مزمنة؟

1.11 هل تم تشخيصك سابقا أو حاليا باضطرابات نفسية؟

1.12 إذا كان الجواب نعم، أذكرها

Part 2: Knowledge about COVID-19:[1]

أجب على الأسئلة التالية بـ"نعم" أو "لا" أو باختيار الجواب الصحيح، و إن لم تعرف الإجابة فاختر "لا أعلم":

2.1 هل المسبب لمرض فيروس كورونا 2019 (COVID-19) معروف؟

2.2 هل يمكن أن ينتقل مرض فيروس كورونا 2019 (COVID-19) بين الناس؟

2.3 هل يمكن أن يصاب الشخص بمرض فيروس كورونا 2019 (COVID-19) من غير أن يتعرض لشخص حامل للفيروس ؟

2.4 هل يمكن أن يحمل الشخص الفيروس المسبب لمرض فيروس كورونا 2019 (COVID-19) من دون أن تظهر عليه أي أعراض؟

2.5 ما هي أعراض الإصابة بمرض فيروس كورونا 2019 (COVID-19) :

-حمى –سعال –عطاس -آلام المفاصل -ضيق في التنفس -تبقع في الجلد –اسهال

-احمرار العينين -احتقان الانف –انعدام حاسة الشم

2.6 هل هناك وسائل تقي من الإصابة بمرض فيروس كورونا 2019 (COVID-19) ؟

2.7 ما هي الوسائل التي ينصح بها للوقاية من انتشار مرض فيروس كورونا 2019 (COVID-19) ؟

-البقاء في المنزل -غسل اليدين بالماء و الصابون -تجنب المصافحة باليد -ارتداء القناع الطبي -التجمع لفترات قصيرة

–تجنب المخالطة اللصيقة بشخص لديه أعراض -استخدام المناديل عند العطس -استخدام القفازات الطبية -تجنب لمس الوجه

2.7 هل توجد حالات لمرضى مصابين بمرض فيروس كورونا 2019 (COVID-19) في المملكة العربية السعودية؟

2.8 هل هناك علاج لمرض فيروس كورونا 2019 (COVID-19) ؟

2.9 هل يعتبر مرض فيروس كورونا 2019 (COVID-19) أكثر إماته من أوبئة العدوى التنفسية السابقة (مثل متلازمة الشرق الأوسط التنفسية أو انفلونزا الخنازير أو أنفلونزا الطيور)؟

2.10 هل يعتبر مرض فيروس كورونا 2019 (COVID-19) معدٍ أكثر من أوبئة العدوى التنفسية السابقة (مثل متلازمة الشرق الأوسط التنفسية أو انفلونزا الخنازير أو أنفلونزا الطيور)؟

Part 3: Depression Anxiety Stress DASS-21:[2]

اقرأ كل من العبارات التالية و اختر الجملة التي تبين درجة انطباق هذا الشعور عليك في الأسبوع الماضي.

لا يوجد إجابات صحيحة أو خاطئة.

لا تقضي وقتا طويلاً في الإجابة على أي منها..

أستعمل التقديرات التالية:

0 : لا ينطبق علي بتاتاً

1 : ينطبق علي بعض الشيء أو قليلاً من الأوقات

2 : ينطبق علي بدرجة ملحوظة أو بعض الأوقات

3: ينطبق علي كثيرا جداً، أو معظم الأوقات

3.1 وجدت صعوبة في الاسترخاء و الراحة

3.2 شعرت بجفاف في حلقي

3.3 لم يبدو لي أن بإمكاني الإحساس بمشاعر إيجابية على الإطلاق

3.4 شعرت بصعوبة في التنفس (شدة التنفس السريع، اللهثان بدون القيام بمجهود جسدي مثلاً)

3.5 وجدت صعوبة في أخذ المبادرة بعمل الأشياء

3.6 كنت أميل إلى ردة فعل مفرطة للظروف و الأحداث

3.7 شعرت برجفة (باليدين مثلاً)

3.8 شعرت بأنني أستهلك الكثير في الطاقة العصبية (شعرت بأنني استهلك الكثير من قدرتي على تحمل التوتر العصبي)

3.9 كنت خائفاً من مواقف قد أفقد فيها السيطرة على أعصابي و أسبب إحراجاً لنفسي

3.10 شعرت بأنني ليس لدي أي شيء أتطلع إليه

3.11 شعرت بأنني مضطرب و منزعج

3.12 أجد صعوبة في الاسترخاء

3.13 شعرت بالحزن و الغم

3.14 كنت لا أستطيع تحمل أي شيء يحول بيني و بين ما أرغب في القيام به

3.15 شعرت بأنني على وشك الوقوع في حالة من الرعب المفاجئ بدون سبب

3.16 فقدت الشعور بالحماس لأي شيء

3.17 شعرت بأن قيمتي قليلة كشخص

3.18 شعرت بأنني أميل إلى الغيظ بسرعة

3.19 شعرت بنبضات قلبي بدون مجهود جسدي (زيادة في معدل الدقات، أو غياب دقة القلب مثلاً)

3.20 شعرت بالخوف بدون أي سبب وجيه

3.21 شعرت بأن الحياة ليس لها معنى

Part 4: Insomnia Severity Index (ISI):[3]

الرجاء الإجابة على الاسئلة التالية باختيار الوصف المناسب لك:

- الرجاء تقييم شدة مشكلة الأرق الخاصة بك حاليا:

0: ليس على الاطلاق

1: خفيف

2: متوسط

3: شديد

4: شديد جداً

1) صعوبة في الاستغراق في النوم

2) صعوبة في البقاء نائماً

ج) مشاكل في الاستيقاظ مبكراً جداً

- كم أنت راضي\غير راضي عن نمط نومك الحالي:

0: راضي جداً   1:   2: راضي بشكل متوسط   3:   4:غير راضي جداً

0: ليس على الاطلاق

1: قليلاً

2: بعض الشيء

3: كثيراً

4: كثيراً جداً

- إلى أي مدى تعتبر أن مشكلة النوم الخاصة بك تتدخل في وظائفك اليومية (مثلا: الإرهاق خلال النهار، القدرة على العمل في وظيفتك\الأعمال اليومية، التركيز، الذاكرة، المزاج، ...الخ):

- كم تظن أن مشكلة النوم الخاصة بك ملحوظة للآخرين من حيث الأضرار بجودة حياتك:

- كم أنت مهتم بمشكلة نومك الحالية:

Part 5: Brief COPE:

العبارات التالية تصف طريقتك في التكيّف منذ بداية الوضع الحالي. الرجاء اختيار الجملة التي تشير إلى أي مدى توافق أو لا توافق على كل عبارة:

1 : أنا لم أفعل هذا على الإطلاق

2 : لقد فعلت هذا قليلاً

3 : لقد كنت أفعل هذا بشكل متوسط

4 : لقد فعلت هذا كثيراً

4.1 لجأت إلى العمل أو الأنشطة الأخرى لتصفية ذهني

4.2 ركّزت جهودي على القيام بشيء حيال الوضع الذي أنا فيه

4.3 كنت أقول لنفسي "هذا ليس حقيقياً"

4.4 استخدمت الكحول أو المخدرات لأشعر على نحو أفضل

4.5 حصلت على الدعم العاطفي من الآخرين

4.6 تخلّيت عن محاولة التعامل مع الوضع

4.7 اتخذت إجراءات في محاولة لجعل الوضع يبدو أفضل

4.8 كنت أرفض أن أصدق أن ما حدث قد حدث فعلاً

4.9 قلت أشياء حتى أتخلص أو أهرب من المشاعر غير السارة المتعلقة بالوضع

4.10 حصلت على المساعدة و المشورة من أشخاص آخرين

4.11 استخدمت الكحول أو المخدرات لتساعدني في الخروج من الوضع

4.12 حاولت أن أرى الوضع في صورة مختلفة، لجعله يبدو أكثر إيجابية

4.13 كنت انتقد نفسي

4.14 حاولت الخروج باستراتيجية حول ما يجب القيام به

4.15 حصلت على الراحة و التفهم من شخص ما

4.16 تخلّيت عن محاولة التأقلم مع الوضع

4.17 بحثت عن شيء جيّد في ما يحدث

4.18 صنعت نكتاً حول هذا الوضع

4.19 فعلت أشياء لأقلل من التفكير في الوضع، مثل الذهاب للسينما أو التسوق أو مشاهدة التلفزيون أو القراءة أو أحلام اليقظة أو النوم

4.20 قبلت بواقع الحقيقة أنه حدث

4.21 كنت أعبر عن مشاعري السلبية

4.22 حاولت أن أجد الراحة في ديني و معتقداتي الروحانية

4.23 حاولت الحصول على مشورة أو مساعدة من الآخرين حول ما يجب القيام به

4.24 تعلمت كيفية العيش مع الوضع

4.25 فكرت مليا في الخطوات التي يجب اتخاذها

4.26 لُمت نفسي على الأشياء التي حدثت

4.27 صليت أو لجأت للتأمل

4.28 كنت أسخر من هذا الوضع

Part 6: Intolerance of Uncertainty Scale (IUS-12):

الرجاء قراءة كل عبارة بعناية و اختيار الجملة التي تصفك بشكل أكثر من غيرها::

1 : لا تنطبق عليّ إطلاقاً

2 : تنطبق عليّ قليلاً

3 : تنطبق عليّ إلى حدّ ما

4 : تنطبق عليّ إلى حد كبير

5 : تنطبق عليّ تماماً

5.1 عندما تحدث أمور ما فجأة، أصبح منزعجاً جداً.

5.2 أنزعج عندما يكون هناك أمورٌ لا أعرفها.

5.3 يجب على الناس دائماً أن يفكروا بما سيحدث مستقبلاً فهذا سوف يمنع حدوث الأمور السيئة.

5.4 حتى لو خططت للأمور بشكل جيد، فإن أمراً واحداً بسيطاً يمكن أن يفسد كل شيء.

5.5 أريد دائماً أن أعرف ماذا سوف يحدث لي في المستقبل.

5.6 لا استطيع تحمل حدوث الأمور فجأةً.

5.7 يجب أن أكون دائماً مستعد للأمور قبل أن تحدث.

5.8 شعوري بعدم التأكد (عدم اليقين) يعيقني عن إنجاز معظم الأمور.

5.9 ينتابني الجمود حينما أكون غير متأكد ممّا ينبغي فعله.

5.10 عندما لا أعلم ما سيحدث لا استطيع فعل الأمور بشكل جيد.

5.11 أبسط هَمٍ يمكن أن يعيقني من فعل الأمور.

5.12 يجب أن أبتعد عن كل الأمور التي لست متأكداً منها.

1. MOH. Novel Coronavirus (COVID-19). https://www.moh.gov.sa/en/HealthAwareness/EducationalContent/PublicHealth/Pages/corona.aspx. Published 2020.
2. Moussa MT, Lovibond P, Laube R, Megahead HA. Psychometric Properties of an Arabic Version of the Depression Anxiety Stress Scales (DASS). *Res Soc Work Pract*. 2017. doi:10.1177/1049731516662916
3. Suleiman KH, Yates BC. Translating the insomnia severity index into Arabic. *J Nurs Scholarsh*. 2011. doi:10.1111/j.1547-5069.2010.01374.x
