# Supplementary material for: Mental health and its association with coping strategies and intolerance of uncertainty during the COVID-19 pandemic among the general population in Saudi Arabia: cross-sectional study
Source: BMC Psychiatry. 2021 Jul 28;21:382. doi: 10.1186/s12888-021-03370-4 (PMC8317145; doi:10.1186/s12888-021-03370-4)
Supplement: Supplementary file 2 — Additional file 2. [file 12888_2021_3370_MOESM2_ESM.zip › Study Questionnaire EnglishR5.pdf]

Study Questionnaire (English Version):

Study Title:

Mental Health and its association with coping strategies and intolerance of uncertainty during the COVID-19 pandemic among the general population in Saudi Arabia.

Part 1: Demographics:

**1. Age**
**2. Gender:**
Male
Female
**3. Marital Status:**
Single
Married
Divorced
Widow
**4. Region of Residence**
Riyadh Region
Makkah Region
Madinah Region
Qassim Region
Eastern Region
Asir Region
Tabouk Region
Ha’il Region
Northern Borders Region
Jazan Region
Najran Region
Albahah Region
Aljouf Region
**5. Occupation Field:**
Student
Education
Healthcare
Security
Military
Trade
Food and Catering
Media
Transportation
Telecommunication and Information
Engineering

Law
Administration
Freelance
Other occupations
Retired
Not working
**6. Contact with patients at work:**
Yes
No
**7. Family member with patient contact at work:**
Yes
No
**8. Had contact with a COVID-19 suspected or confirmed case:**
Yes
No
Unknown
**9. Chronic Medical Illnesses:**
Yes
No
**10. Diagnosed Mental Illness:**
Yes
No
**11. Categories of Mental Illness:**
Depressive Disorders
Anxiety Disorders
Neurodevelopmental Disorder
Psychotic Disorders
Bipolar Disorders
Obsessive Compulsive Disorder
Trauma Related Disorders
Eating Disorders
Sleep Disorders
Personality Disorders
Other Disorders

Part 2: Knowledge about COVID-19:

**12. Is the cause for COVID-19 known?**
Yes
No
I do not know
**13. Can COVID-19 be transmitted between people?**
Yes
No

I do not know
**14. Can a person have COVID-19 without having had contact with someone carrying the virus?**
Yes
No
I do not know
**15. Can a person be carrying the virus causing COVID-19 without having any symptoms?**
Yes
No
I do not know
**16. What are the symptoms of having COVID-19? [Choose the correct answer/s]**
Fever
Cough
Sneeze
Stuffy Nose
Shortness of Breath
Skin Discoloration
Diarrhea
Red Eye
Joint Pain
Anosmia
**17. What are the recommended methods for preventing the spread of COVID-19? [Choose the correct answer/s]**
Wearing medical gloves
Not touching one's face
Staying at home
Washing hands with water and soap
Not shaking hands

Wearing medical mask
Using tissues when sneezing
Gathering for short durations
Avoiding close contact with someone with symptoms
**18. Are there cases of COVID-19 in Saudi Arabia?**
Yes
No
I do not know
**19. Is there treatment for COVID-19?**
Yes
No
I do not know
**20. Is COVID-19 considered deadlier than previous respiratory infection epidemics (such as MERS, Swine Flu, or Bird Flu)?**
Yes
No
I do not know
**21. Is COVID-19 considered more infectious than previous respiratory infection epidemics (such as MERS, Swine Flu, or Bird Flu)?**
Yes
No
I do not know

Part 3: DASS-21:

Please read each statement and circle a number 0, 1, 2 or 3 which indicates how much the statement applied to you **over the past week**. There are no right or wrong answers. Do not spend too much time on any statement.
The rating scale is as follows:
0 Did not apply to me at all
1 Applied to me to some degree, or some of the time
2 Applied to me to a considerable degree or a good part of time
3 Applied to me very much or most of the time

1 (s)	**I found it hard to wind down**	0	1	2	3
2 (a)	**I was aware of dryness of my mouth**	0	1	2	3
3 (d)	**I couldn’t seem to experience any positive feeling at all**	0	1	2	3

4 (a)	**I experienced breathing difficulty (e.g. excessively rapid breathing, breathlessness in the absence of physical exertion)**	0	1	2	3
5 (d)	**I found it difficult to work up the initiative to do things**	0	1	2	3
6 (s)	**I tended to over-react to situations**	0	1	2	3
7 (a)	**I experienced trembling (e.g. in the hands)**	0	1	2	3
8 (s)	**I felt that I was using a lot of nervous energy**	0	1	2	3
9 (a)	**I was worried about situations in which I might panic and make a fool of myself**	0	1	2	3
10 (d)	**I felt that I had nothing to look forward to**	0	1	2	3
11 (s)	**I found myself getting agitated**	0	1	2	3
12 (s)	**I found it difficult to relax**	0	1	2	3
13 (d)	**I felt down-hearted and blue**	0	1	2	3
14 (s)	**I was intolerant of anything that kept me from getting on with what I was doing**	0	1	2	3
15 (a)	**I felt I was close to panic**	0	1	2	3
16 (d)	**I was unable to become enthusiastic about anything**	0	1	2	3
17 (d)	**I felt I wasn't worth much as a person**	0	1	2	3
18 (s)	**I felt that I was rather touchy**	0	1	2	3
19 (a)	**I was aware of the action of my heart in the absence of physical exertion (e.g. sense of heart rate increase, heart missing a beat)**	0	1	2	3
20 (a)	**I felt scared without any good reason**	0	1	2	3
21 (d)	**I felt that life was meaningless**	0	1	2	3

Part: 4: Brief COPE:

1 = I haven't been doing this at all
2 = I've been doing this a little bit
3 = I've been doing this a medium amount
4 = I've been doing this a lot

**1. I've been turning to work or other activities to take my mind off things.**	1	2	3	4
**2. I've been concentrating my efforts on doing something about the situation I'm in.**	1	2	3	4

**3. I've been saying to myself "this isn't real.".**	1	2	3	4
**4. I've been using alcohol or other drugs to make myself feel better.**	1	2	3	4
**5. I've been getting emotional support from others.**	1	2	3	4
**6. I've been giving up trying to deal with it.**	1	2	3	4
**7. I've been taking action to try to make the situation better.**	1	2	3	4
**8. I've been refusing to believe that it has happened.**	1	2	3	4
**9. I've been saying things to let my unpleasant feelings escape.**	1	2	3	4
**10. I’ve been getting help and advice from other people.**	1	2	3	4
**11. I've been using alcohol or other drugs to help me get through it.**	1	2	3	4
**12. I've been trying to see it in a different light, to make it seem more positive.**	1	2	3	4
**13. I’ve been criticizing myself.**	1	2	3	4
**14. I've been trying to come up with a strategy about what to do.**	1	2	3	4
**15. I've been getting comfort and understanding from someone.**	1	2	3	4
**16. I've been giving up the attempt to cope.**	1	2	3	4
**17. I've been looking for something good in what is happening.**	1	2	3	4
**18. I've been making jokes about it.**	1	2	3	4
**19. I've been doing something to think about it less, such as going to movies, watching TV, reading, daydreaming, sleeping, or shopping.**	1	2	3	4
**20. I've been accepting the reality of the fact that it has happened.**	1	2	3	4
**21. I've been expressing my negative feelings.**	1	2	3	4
**22. I've been trying to find comfort in my religion or spiritual beliefs.**	1	2	3	4
**23. I’ve been trying to get advice or help from other people about what to do.**	1	2	3	4
**24. I've been learning to live with it.**	1	2	3	4
**25. I've been thinking hard about what steps to take.**	1	2	3	4
**26. I’ve been blaming myself for things that happened.**	1	2	3	4
**27. I've been praying or meditating.**	1	2	3	4
**28. I've been making fun of the situation.**	1	2	3	4

Part 5: Intolerance of Uncertainty:

1: Not at all characteristic of me

2: A little characteristic of me

3: Somewhat characteristic of me

4: Very characteristic of me

5: Entirely characteristic of me

**1. Unforeseen events upset me greatly.**	1	2	3	4	5
**2. It frustrates me not having all the information I need.**	1	2	3	4	5
**3. Uncertainty keeps me from living a full life.**	1	2	3	4	5
**4. One should always look ahead so as to avoid surprises.**	1	2	3	4	5
**5. A small unforeseen event can spoil everything, even with the best of planning.**	1	2	3	4	5
**6. When it's time to act, uncertainty paralyses me.**	1	2	3	4	5
**7. When I am uncertain I can't function very well.**	1	2	3	4	5
**8. I always want to know what the future has in store for me.**	1	2	3	4	5
**9. I can't stand being taken by surprise.**	1	2	3	4	5
**10. The smallest doubt can stop me from acting.**	1	2	3	4	5
**11. I should be able to organize everything in advance.**	1	2	3	4	5
**12. I must get away from all uncertain situations.**	1	2	3	4	5
